# Supplementary material for: Training Internal Medicine Residents to Perform Telemedicine Visits: A Novel Skill-Based Curriculum
Source: MedEdPORTAL. 2025 Jul 8;21:11540. doi: 10.15766/mep_2374-8265.11540 (PMC12237798; doi:10.15766/mep_2374-8265.11540)
Supplement: Supplementary file 1 — Faculty Facilitator Guide.docxResident Handout.docxDirect Observation Checklist.docxTelehealth Faculty Development Session.pptxPre- and Posttest.docx [file mep_2374-8265.11540-s001.zip › A. Faculty Facilitator Guide.docx]

**Part 1: Facilitator Instructions for Small Group Case-based Discussion**

**Session Overview**

This interactive session is designed to improve confidence with telemedicine skills for residents who are familiar with telemedicine technology and use. The content focuses on three Telehealth Competencies from the American Association of Medical Colleges (AAMC), Patient Safety and Appropriate Use by introducing and practicing a framework for telemedicine triage, Data Collection and Assessment through a physical examination role play, and Communication through discussion of “webside” manner and telemedicine communication skills.^1^ This session will be delivered through small group case-based discussion and can be delivered in a 45minute session.

**Session Objectives**

By the end of the activity, the learners will be able to:

1. Identify clinical scenarios that are most appropriate for different visit types, including phone, video, and in-person.
2. Perform a focused history and physical exam through a telemedicine visit.
3. Demonstrate essential skills necessary for efficient, timely telemedicine visits, including disease management, counseling, follow-up, technology use, and presenting to a clinical preceptor.
4. Name 3 best practices for effective communication during a telemedicine visit.

**Session Outline**

| Time | Topic |
| --- | --- |
| 3 min | Introduction |
| 5 min | Case 1 Visit Triage |
| 5 min | Case 2 Visit Triage |
| 10 min | Case 3 Visit Triage and Physical Exam Role Play |
| 5 min | Case 3 Communication Skills |
| 5 min | Case 3 Follow-up Planning |
| 5 min | Case 4 Communication Skills |
| 4 min | Case 4 Visit Triage |
| 3 min | Wrap-up |

**Session Delivery**

Invite residents to take turns reading the case (grey-shaded portion) and subsequent questions aloud. Suggested facilitator responses are shown in italics on the facilitator guide.

Table. Numbered Cases with Descriptions Anchored to Telemedicine Skills, Educational Objectives, and AAMC Telehealth Domains.

| **Numbered Case Descriptions.** | **Skill** | **Educational Objective** | **AAMC Telehealth Domain** |
| --- | --- | --- | --- |
| 1. 1) Follow-up visit for depression. | Triage to visit type. | Identify clinical scenarios that are most appropriate for different visit types, including phone, video, and in-person. | Patient Safety and Appropriate Use of Telehealth |
| 1. 2) New acute concern for severe fatigue and shortness of breath. | Triage to visit type. | Identify clinical scenarios that are most appropriate for different visit types, including phone, video, and in-person. | Patient Safety and Appropriate Use of Telehealth |
| 1. 3) New acute concern for shoulder pain. | Triage to visit type. | Identify clinical scenarios that are most appropriate for different visit types, including phone, video, and in-person. | Patient Safety and Appropriate Use of Telehealth |
| Evaluate shoulder pain with physical exam. | Adapt shoulder exam to a video visit. | Perform a focused history and physical exam through a telemedicine visit. | Data Collection and Assessment Via Telehealth |
| Prepare for a video visit. | Define “webside” manner. | Name 3 best practices for effective communication during a telemedicine visit. | Communication via Telehealth |
| Communicate follow-up plan. | Use telemedicine work-flows. | Demonstrate essential skills necessary for efficient, timely telemedicine visits, including disease management, counseling, follow-up, technology use, and presenting to clinical preceptor. | Patient Safety and Appropriate Use of Telehealth |
| 4) Patient who needs re-direction. | “Triple E” strategy for interruptions to set an agenda. | Name 3 best practices for effective communication during a telemedicine visit. | Communication via Telehealth |
| New acute concern requiring triage to in-person evaluation. | Triage to visit type. | Identify clinical scenarios that are most appropriate for different visit types, including phone, video, and in-person. | Patient Safety and Appropriate Use of Telehealth |

**A Framework for Telemedicine Visits**

**Faculty Facilitator Guide**

By the end of the activity, the learners will be able to:

1. Identify clinical scenarios that are most appropriate for different visit types, including phone, video, and in-person.
2. Perform a focused history and physical exam through a telemedicine visit.
3. Demonstrate essential skills necessary for efficient, timely telemedicine visits, including disease management, counseling, follow-up, technology use, and presenting to a clinical preceptor.
4. Name 3 best practices for effective communication during a telemedicine visit.

**Introduction** (3min)

In response to the COVID-19 pandemic, synchronous telemedicine visits rapidly became part of primary care with a need for telemedicine skill development. Subsequently, formal competencies and milestones in telemedicine care are recommended for physicians and internal medicine residents.^1,2^ In this case-based discussion, we will cover Patient Safety and Appropriate Use of Telehealth by introducing and practicing a framework for telemedicine triage, Data Collection and Assessment via Telehealth through a physical examination role play, and Communication via Telehealth through discussion of “webside” manner and telemedicine communication skills

We will start with triage of patients to appropriate visit types.

While your schedulers may often make these decisions, you will likely have patient messages that prompt you to decide what visit types allow you to care for a patient’s concern. Additionally, when planning follow-up after a visit, you should decide what visit type is appropriate for the next visit.

We recommend a three-step strategy to assist you in making these decisions:

1. What is my differential diagnosis for the patient’s presenting concern? Given the differential diagnosis, are there any safety issues that would require an in-person or face to face visit?
2. How would an in-person physical exam change my evaluation of this patient?
3. What visit types (telephone, video, or in-person) are accessible to this patient?

Please use this strategy in the next few cases.

(5min)

**Case 1:** You just finished an in-person visit for Mr. Smith, a 46-year-old man with depression and anxiety. He had difficulty sleeping and increased anxiety. He does not experience suicidal or homicidal ideation. TSH was recently checked and normal. He is low risk for obstructive sleep apnea. You increased his dose of escitalopram to 20mg and recommended increasing his therapy sessions to every other week. He is agreeable to this plan.

You want to schedule a follow-up visit with him to see if your changes are effective when you are back on your next clinic block.

**What type of visit would you recommend?**

1. **What is my differential diagnosis for the patient’s presenting concern? Given the differential diagnosis, are there any patient safety issues that would require an in-person visit?**

*Consider what you suspect is happening to this patient. Likely his depression and anxiety are not well controlled at present, and poor sleep is a symptom of this. Hyperthyroidism and sleep apnea are unlikely. There are no obvious patient safety issues.*

*The oft quoted medical teaching that “90% of the diagnosis comes from the history and 10% of the diagnosis come from the physical exam” will be applied in decision-making about triaging patients to telemedicine vs. in-person visits and directly affects the next question in this framework.*

1. **How would an in-person physical exam change my evaluation of this patient?**

*At this point, an in-person physical exam is unlikely to change the management of this patient’s care.*

1. **What are the possible visit options for this patient?**

***He could follow-up as telephone, video, or in person visit.***

*To finalize your recommendation, next steps are to assess:*

- *If he has suitable technology and ability to participate in a video or telephone visit*
- *If he has barriers to in-person visits such as transportation issues*

(5min)

**Case 2:** Ms. Yang is a 35-year-old woman who has allergic asthma and is on birth control for a history of menorrhagia. She called your nurse with concern for severe fatigue and new shortness of breath over the past 2 weeks. You have openings on your afternoon schedule and want her to be scheduled with you.

**What type of visit would you recommend?**

1. **What is my differential diagnosis for the patient’s presenting concern? Given the differential diagnosis, are there any patient safety issues that would require an in-person visit?**

*The differential diagnosis for this patient may include symptomatic anemia, pulmonary embolism, uncontrolled asthma, acute asthma exacerbation or anxiety disorder.*

*History alone is unlikely to reveal the etiology of the patient’s fatigue and shortness of breath, so a physical exam is needed. Given the potentially life-threatening conditions on your differential diagnosis,* ***it is unsafe to evaluate the patient through telemedicine video or telephone visit only.***

***Follow-up question: What other patient concerns are likely to require in-person evaluation?***

*Ask residents to generate a list of patient concerns that should be triaged to in-person visits. These may include:*

- *Altered mental status*
- *Shortness of breath, palpitations, chest pain*
- *Abdominal pain*
- *Vaginal or genital concerns*
- *Suicidal or homicidal ideation*
- *Syncope or presyncope*

1. **How would an in-person physical exam change my evaluation of this patient?**

***This patient requires an in-person exam*** *to narrow your differential diagnosis. The exam would include but not be limited to: vital signs to evaluate for tachycardia/hypotension or hypoxia, auscultation of her lungs for wheezing, inspection for pallor, boggy nasal mucosa and posterior oropharynx cobble-stoning. She may also require a vaginal exam if having menorrhagia to look for active bleeding or palpate for an enlarged uterus.*

1. **What are the possible visit options for this patient?**

*She should be triaged to an in-person visit given these safety issues.*

*If she cannot attend an in-person visit, a video visit is preferred over a telephone visit as it would allow visual inspection of respiratory distress or accessory muscle use. You could then triage her to the emergency room if needed.*

(10min)

**Case 3:** Mr. Ayer is a 58-year-old man with hypertension and chronic obstructive pulmonary disease. He is calling in with 1 week of shoulder pain and wants to know what to do about it. You have openings in your afternoon schedule and want him to have an appointment.

**What type of visit would you recommend?**

1. **What is my differential diagnosis for the patient’s presenting concern? Given the differential diagnosis, are there any patient safety issues that would require an in-person visit?**

*The differential diagnosis includes but is not limited to rotator cuff tendinopathy, adhesive capsulitis, labrum tear, acromial-clavicular arthritis, gleno-humeral arthritis. There are no major patient safety issues given this differential diagnosis.*

1. **How would an in-person physical exam change my evaluation of this patient?**

*The physical exam will be important in evaluating this patient’s concern. Given the musculoskeletal exam primarily requires the ability to inspect the joint, it is amenable to physical exam via a video.*

**What considerations are needed when performing video visit physical exam?**

- *Camera positioning matters.*
- *Expose body parts if needed (maintaining patient modesty).*
- *Patients can palpate their own body.*
- *Patients may be able to use the contralateral side to do things that you would have performed in clinic (ie assist in passive ROM, perform Neer test).*
- *Demonstrating a maneuver may be more helpful that describing it.*

***Preceptors: Ask 2 residents to ROLE PLAY adapting the physical exam for shoulder pain to a video visit.***

***One resident is the DOCTOR on a video visit.***

***One resident is the PATIENT on a video visit.***

*The goal is for residents to think about how to adapt physical exam to the video – not to test their knowledge of the shoulder exam.*

*Remind/coach the DOCTOR to perform 2 of the exam maneuvers (OR any others that they choose to do OR that you prefer to coach them to do).*

1. *Range of Motion (ROM): Perform flexion/extension, abduction/adduction, internal/external rotation.*
   1. *If active ROM is limited, can the patient complete passive ROM using their contralateral arm to assist?*
2. *“Neer Test for Impingement”: The arm is fully pronated and then flexed while the examiner or patient’s contralateral arm presses down on the shoulder to provide resistance. (If pain, subacromial impingement is present.)*
3. *“Off-back Test” or ”Lift-off Test”: Shoulder is internally rotated. Dorsum of the hand rests on mid-lumbar spine. Patient is instructed to lift the hand off the back posteriorly. (If pain or weakness, there is likely subscapularis injury.)*

*[As the preceptor, if you would like to watch a video visit adaptation of the shoulder exam to prepare for teaching the conference, Stanford developed an educational resource for several physical exam maneuvers. To review the shoulder exam, start at 11:06 and watch for approximately 5 minutes.^3^* ***Please do not show this during the session.****]*

***Follow-up questions:***

***What findings would be worrisome for an acute rotator cuff tear such that you would refer for urgent orthopedic evaluation?***

*Weakness rather than pain limiting the range of motion or other exam maneuvers.*

***Take a few minutes to consider how to adapt other physical exam maneuvers to the video visit.***

*Examples:*

*Respiratory exam: How many word dyspnea does the patient have? Are they using accessory muscles to breathe? Is there audible wheeze?*

*Abdominal exam: Stomping the foot on the floor without doubling over argues against peritonitis. Can the patient palpate where the pain is the worst? Can you coach them to perform the exam for Murphy’s sign?*

1. **What are the possible visit options for this patient?**

*If he is assessed to not have any technological barriers, he could do an in-person, video, or telephone visit. Patient preference should also be considered, and he requests a video visit.*

(5min)

**Case 3 Continued**

Mr. Ayer is scheduled for a video visit to evaluate his shoulder. You have only met Mr. Ayer once previously when addressing his chronic illnesses. He had a great relationship with his prior resident physician, and you did not feel that you developed a strong rapport with him yet.

1. **Briefly describe best practices for communicating with patients over telemedicine so that you demonstrate a good “webside” manner.**
2. *Virtual room set up*
   1. *Make sure lighting is adequate and allows the patient to see you.*
   2. *Ok to take a mask off if you were wearing one.*
   3. *Professional dress – especially if you’re working from home*
   4. *Call the patient if they have not joined the video visit as they may be having technology issues*
   5. *Address any privacy concerns – for example, if patient has others around them, or if you are not in a private location*
3. *Connecting with patient during the visit*
   1. *Mimic eye contact by occasionally looking directly into the camera*
   2. *Use non-verbal cues to facilitate communication: smiling, head nodding, leaning in*
   3. *Avoid distractions – turn cell phone off, turn off email alerts, etc*
   4. *If needed, acknowledge that communication over telemedicine may feel awkward.*
   5. *Observe your patient’s environment. Consider inviting the patient to briefly share something about the environment.*

(5min)

**Case 3 continued**

You diagnose Mr. Ayer with rotator cuff tendinopathy and include physical therapy in your recommendations for his care. You would like to follow-up with him in 6 weeks to see his response. Before your next visit, you also want him to complete his annual creatinine and potassium labs as he is on hydrochlorothiazide for his hypertension.

1. **How will you advise him to follow through on your recommendations and schedule a follow-up appointment?**

*Preceptors, please advise residents on institutional or site-specific recommendations for how to order testing or complete referrals during telemedicine encounters if these work-flows are different from in-person visits.*

(5min)

**Case 4:** Mrs. Apple is a 64-year-old woman with diabetes seen for a video visit to discuss worsening of her seasonal allergies. She is excited about the technology and is delighted to introduce you to her pet dog and to show off her collection of teapots. She hasn’t looked back at the video camera in several minutes. While you enjoy learning more about her and appreciate your positive rapport, you want to be sure to address her medical concerns and use the time efficiently.

1. **What communication tools can be used to redirect this patient?**

*Interruption and agenda setting can be helpful. One approach is below.*

*Dr. Larry Mauksch, medical communication researcher, offers the* ***“Triple E” strategy*** *for interruptions*

1. ***Excuse*** *yourself, acknowledging the interruption.*
2. ***Empathize*** *about the topic being interrupted.*
3. *E****xplain*** *the reason for the interruption.*

*As an example, he combines this with the “Anything else question?” for agenda setting,^4^*

*“Excuse me for a moment. Your knee has been painful. Before we talk further about this pain, I’d like to know if you have something else important to address today. This way you and I can figure out how to make the best use of our time.”*

(4min)

**Case 4 Continued**

You set an agenda for the visit, which includes discussion of Mrs. Apple’s seasonal allergies and review of her diabetes. For her seasonal allergies, you start Mrs. Apple on fluticasone.

You review her diabetes care and add empagliflozin 10mg to metformin 1000mg BID because the A1C she recently had checked was 8.8%. Her next visit is in 3 months. You plan to see her in person to check her A1C and complete her foot exam and retinal imaging.

Although you set an agenda at the beginning of the visit, she does say, “Doc, while I have you on the video, I did have one more thing to discuss please.”

She’s having trouble going up her steps at home, and she has not left her house as a result. She no longer walks a mile a few times per week. Yesterday, she was so winded while unpacking groceries (walking from the end of the driveway to her basement garage and up the stairs to the kitchen) that she had to rest twice. She asks, “Am I just out of shape because I haven’t been exercising?”

1. **How will you respond to Mrs. Apple’s new concern?**

*Mrs. Apple is describing new dyspnea on exertion.* ***Residents should use the 3-step approach discussed earlier in the module.*** *The differential diagnosis does include deconditioning as she mentioned, but also includes many other diagnoses with safety issues such as unstable angina, new onset heart failure, pulmonary embolism, etc. Recognizing these safety issues, she should be re-triaged from a video visit to an urgent in-person visit for a complete evaluation. Despite reasonable initial triage, patients may need to be evaluated in-person if the video or telephone visit is no longer appropriate.* ***Mrs. Apples is not having chest pain or shortness of breath at present. You recommend that she be seen in clinic with you later that afternoon, and she agrees.***

**Take-home Points** (2min)

- When triaging patients, use the 3-step approach introduced in this module:

1. What is my differential diagnosis for the patient’s presenting concern? Given the differential diagnosis, are there any safety issues that would require an in-person or face to face visit?
2. How would an in-person physical exam change my evaluation of this patient?
3. What visit types (telephone, video, or in-person) are accessible to this patient?

- Many aspects of the physical exam can be adapted to telemedicine video visits. In your video visits, practice this skill. If you would like to learn more, Stanford has posted some useful videos.^3^
- We discussed 3 keys to communicating with patients effectively over telemedicine visits:

1. Set up your virtual room – lighting, unmask, dress professionally, address privacy concerns.
2. Communication skills to help connect with patient – eye contact over video, use of nonverbal communication skills, invite patient to share something about environment.
3. Communication skills for interruption and redirection – set an agenda; Triple E strategy of excuse, empathize, explain.^4^

**Wrap-Up** (1min)

Telemedicine is a rapidly evolving mechanism for delivering high-quality patient care. Many aspects of telemedicine are beyond the scope of this curriculum and vary widely based on the local institution. These include but are not limited to federal reimbursement of telemedicine services, billing and coding requirements for telemedicine services, state regulation of telemedicine services, professional society ethical recommendations for telemedicine, institutional capacity for telemedicine, and trouble-shooting the technology. This curriculum introduces concepts of using telemedicine in resident clinic and provides a framework for providing high-quality patient care.

Optional References

1. AAMC. *Telehealth Competencies Across the Learning Continuum*. 2021. *AAMC New and Emerging Areas in Medicine Series*.

2. ACGME. *Internal Medicine Milestones*. 2021. <https://www.acgme.org/globalassets/PDFs/Milestones/InternalMedicineMilestones.pdf>

3. Srinvasan MaA, M. How to Administer a Virtual Physical Exam. Accessed April 30, 2020. <http://medicine.stanford.edu/news/current-news/standard-news/virtual-physical-exam.html>

4. Mauksch LB. Questioning a Taboo: Physicians' Interruptions During Interactions With Patients. *JAMA*. May 5 2020;323(17):1704-1705. doi:10.1001/jama.2020.2980
