# Supplementary material for: Training Internal Medicine Residents to Perform Telemedicine Visits: A Novel Skill-Based Curriculum
Source: MedEdPORTAL. 2025 Jul 8;21:11540. doi: 10.15766/mep_2374-8265.11540 (PMC12237798; doi:10.15766/mep_2374-8265.11540)
Supplement: Supplementary file 1 — Faculty Facilitator Guide.docxResident Handout.docxDirect Observation Checklist.docxTelehealth Faculty Development Session.pptxPre- and Posttest.docx [file mep_2374-8265.11540-s001.zip › B. Resident Handout.docx]

**Part 1: Instructions for Small Group Case-based Discussion**

**Session Overview**

This interactive session is designed to improve confidence with telemedicine skills for residents who are familiar with telemedicine technology and use. The content focuses on three Telehealth Competencies from the American Association of Medical Colleges (AAMC), Patient Safety and Appropriate Use by introducing and practicing a framework for telemedicine triage, Data Collection and Assessment through a physical examination role play, and Communication through discussion of “webside” manner and telemedicine communication skills.^1^ This session will be delivered through small group case-based discussion and can be delivered in a 45minute session.

**Session Objectives**

By the end of the activity, the learners will be able to:

1. Identify clinical scenarios that are most appropriate for different visit types, including phone, video, and in-person.
2. Perform a focused history and physical exam through a telemedicine visit.
3. Demonstrate essential skills necessary for efficient, timely telemedicine visits, including disease management, counseling, follow-up, technology use, and presenting to a clinical preceptor.
4. Name 3 best practices for effective communication during a telemedicine visit.

**Session Outline**

| Time | Topic |
| --- | --- |
| 3 min | Introduction |
| 5 min | Case 1 Visit Triage |
| 5 min | Case 2 Visit Triage |
| 10 min | Case 3 Visit Triage and Physical Exam Role Play |
| 5 min | Case 3 Communication Skills |
| 5 min | Case 3 Follow-up Planning |
| 5 min | Case 4 Communication Skills |
| 4 min | Case 4 Visit Triage |
| 3 min | Wrap-up |

**Session Delivery**

Invite residents to take turns reading the case (grey-shaded portion) and subsequent questions aloud. Suggested facilitator responses are shown in italics on the facilitator guide.

**A Framework for Telemedicine Visits**

**Resident Hand-out**

By the end of the activity, the learners will be able to:

1. Identify clinical scenarios that are most appropriate for different visit types, including phone, video, and in-person.
2. Perform a focused history and physical exam through a telemedicine visit.
3. Demonstrate essential skills necessary for efficient, timely telemedicine visits, including disease management, counseling, follow-up, technology use, and presenting to a clinical preceptor.
4. Name 3 best practices for effective communication during a telemedicine visit.

**Introduction**

In response to the COVID-19 pandemic, synchronous telemedicine visits rapidly became part of primary care with a need for telemedicine skill development. Subsequently, formal competencies and milestones in telemedicine care are recommended for physicians and internal medicine residents.^1,2^ In this case-based discussion, we will cover Patient Safety and Appropriate Use of Telehealth by introducing and practicing a framework for telemedicine triage, Data Collection and Assessment via Telehealth through a physical examination role play, and Communication via Telehealth through discussion of “webside” manner and telemedicine communication skills

We will start with triage of patients to appropriate visit types.

While your schedulers may often make these decisions, you will likely have patient messages that prompt you to decide what visit types allow you to care for a patient’s concern. Additionally, when planning follow-up after a visit, you should decide what visit type is appropriate for the next visit.

We recommend a three-step strategy to assist you in making these decisions:

1. What is my differential diagnosis for the patient’s presenting concern? Given the differential diagnosis, are there any safety issues that would require an in-person or face to face visit?
2. How would an in-person physical exam change my evaluation of this patient?
3. What visit types (telephone, video, or in-person) are accessible to this patient?

Please use this strategy in the next few cases.

**Case 1:** You just finished an in-person visit for Mr. Smith, a 46-year-old man with depression and anxiety. He had difficulty sleeping and increased anxiety. He does not experience suicidal or homicidal ideation. TSH was recently checked and normal. He is low risk for obstructive sleep apnea. You increased his dose of escitalopram to 20mg and recommended increasing his therapy sessions to every other week. He is agreeable to this plan.

You want to schedule a follow-up visit with him to see if your changes are effective when you are back on your next clinic block.

**What type of visit would you recommend?**

1. **What is my differential diagnosis for the patient’s presenting concern? Given the differential diagnosis, are there any patient safety issues that would require an in-person visit?**
2. **How would an in-person physical exam change my evaluation of this patient?**
3. **What are the possible visit options for this patient?**

**Case 2:** Ms. Yang is a 35-year-old woman who has allergic asthma and is on birth control for a history of menorrhagia. She called your nurse with concern for severe fatigue and new shortness of breath over the past 2 weeks. You have openings on your afternoon schedule and want her to be scheduled with you.

**What type of visit would you recommend?**

1. **What is my differential diagnosis for the patient’s presenting concern? Given the differential diagnosis, are there any patient safety issues that would require an in-person visit?**

***Follow-up question: What other patient concerns are likely to require in-person evaluation?***

1. **How would an in-person physical exam change my evaluation of this patient?**
2. **What are the possible visit options for this patient?**

**Case 3:** Mr. Ayer is a 58-year-old man with hypertension and chronic obstructive pulmonary disease. He is calling in with 1 week of shoulder pain and wants to know what to do about it. You have openings in your afternoon schedule and want him to have an appointment.

**What type of visit would you recommend?**

1. **What is my differential diagnosis for the patient’s presenting concern? Given the differential diagnosis, are there any patient safety issues that would require an in-person visit?**
2. **How would an in-person physical exam change my evaluation of this patient?**

**What considerations are needed when performing video visit physical exam?**

***Preceptors: Ask 2 residents to ROLE PLAY adapting the physical exam for shoulder pain to a video visit.***

1. *Range of Motion (ROM): Perform flexion/extension, abduction/adduction, internal/external rotation.*
   1. *If active ROM is limited, can the patient complete passive ROM using their contralateral arm to assist?*
2. *“Neer Test for Impingement”: The arm is fully pronated and then flexed while the examiner or patient’s contralateral arm presses down on the shoulder to provide resistance. (If pain, subacromial impingement is present.)*
3. *“Off-back Test” or ”Lift-off Test”: Shoulder is internally rotated. Dorsum of the hand rests on mid-lumbar spine. Patient is instructed to lift the hand off the back posteriorly. (If pain or weakness, subscapularis injury.)*

***Follow-up questions:***

***What findings would be worrisome for an acute rotator cuff tear such that you would refer for urgent orthopedic evaluation?***

***Take a few minutes to consider how to adapt other physical exam maneuvers to the video visit.***

1. **What are the possible visit options for this patient?**

**Case 3 Continued**

Mr. Ayer is scheduled for a video visit to evaluate his shoulder. You have only met Mr. Ayer once previously when addressing his chronic illnesses. He had a great relationship with his prior resident physician, and you did not feel that you developed a strong rapport with him yet.

1. **Briefly describe best practices for communicating with patients over telemedicine so that you demonstrate a good “webside” manner.**

**Case 3 continued**

You diagnose Mr. Ayer with rotator cuff tendinopathy and include physical therapy in your recommendations for his care. You would like to follow-up with him in 6 weeks to see his response. Before your next visit, you also want him to complete his annual creatinine and potassium labs as he is on hydrochlorothiazide for his hypertension.

1. **How will you advise him to follow through on your recommendations and schedule a follow-up appointment?**

**Case 4:** Mrs. Apple is a 64-year-old woman with diabetes seen for a video visit to discuss worsening of her seasonal allergies. She is excited about the technology and is delighted to introduce you to her pet dog and to show off her collection of teapots. She hasn’t looked back at the video camera in several minutes. While you enjoy learning more about her and appreciate your positive rapport, you want to be sure to address her medical concerns and use the time efficiently.

1. **What communication tools can be used to redirect this patient?**

**Case 4 Continued**

You set an agenda for the visit, which includes discussion of Mrs. Apple’s seasonal allergies and review of her diabetes. For her seasonal allergies, you start Mrs. Apple on fluticasone.

You review her diabetes care and add empagliflozin 10mg to metformin 1000mg BID because the A1C she recently had checked was 8.8%. Her next visit is in 3 months. You plan to see her in person to check her A1C and complete her foot exam and retinal imaging.

Although you set an agenda at the beginning of the visit, she does say, “Doc, while I have you on the video, I did have one more thing to discuss please.”

She’s having trouble going up her steps at home, and she has not left her house as a result. She no longer walks a mile a few times per week. Yesterday, she was so winded while unpacking groceries (walking from the end of the driveway to her basement garage and up the stairs to the kitchen) that she had to rest twice. She asks, “Am I just out of shape because I haven’t been exercising?”

1. **How will you respond to Mrs. Apple’s new concern?**

**Take-home Points**

- When triaging patients, use the 3-step approach introduced in this module:

1. What is my differential diagnosis for the patient’s presenting concern? Given the differential diagnosis, are there any safety issues that would require an in-person or face to face visit?
2. How would an in-person physical exam change my evaluation of this patient?
3. What visit types (telephone, video, or in-person) are accessible to this patient?

- Many aspects of the physical exam can be adapted to telemedicine video visits. In your video visits, practice this skill. If you would like to learn more, Stanford has posted some useful videos.^3^
- We discussed 3 keys to communicating with patients effectively over telemedicine visits:

1. Set up your virtual room – lighting, unmask, dress professionally, address privacy concerns.
2. Communication skills to help connect with patient – eye contact over video, use of nonverbal communication skills, invite patient to share something about environment.
3. Communication skills for interruption and redirection – set an agenda; Triple E strategy of excuse, empathize, explain.^4^

**Wrap-Up**

Telemedicine is a rapidly evolving mechanism for delivering high-quality patient care. Many aspects of telemedicine are beyond the scope of this curriculum and vary widely based on the local institution. These include but are not limited to federal reimbursement of telemedicine services, billing and coding requirements for telemedicine services, state regulation of telemedicine services, professional society ethical recommendations for telemedicine, institutional capacity for telemedicine, and trouble-shooting the technology. This curriculum introduces concepts of using telemedicine in resident clinic and provides a framework for providing high-quality patient care.

Optional References

1. AAMC. *Telehealth Competencies Across the Learning Continuum*. 2021. *AAMC New and Emerging Areas in Medicine Series*.

2. ACGME. *Internal Medicine Milestones*. 2021. <https://www.acgme.org/globalassets/PDFs/Milestones/InternalMedicineMilestones.pdf>

3. Mauksch LB. Questioning a Taboo: Physicians' Interruptions During Interactions With Patients. *JAMA*. May 5 2020;323(17):1704-1705. doi:10.1001/jama.2020.2980

4. Srinvasan MaA, M. How to Administer a Virtual Physical Exam. Accessed April 30, 2020. <http://medicine.stanford.edu/news/current-news/standard-news/virtual-physical-exam.html>
