# Supplementary material for: Training Internal Medicine Residents to Perform Telemedicine Visits: A Novel Skill-Based Curriculum
Source: MedEdPORTAL. 2025 Jul 8;21:11540. doi: 10.15766/mep_2374-8265.11540 (PMC12237798; doi:10.15766/mep_2374-8265.11540)
Supplement: Supplementary file 1 — Faculty Facilitator Guide.docxResident Handout.docxDirect Observation Checklist.docxTelehealth Faculty Development Session.pptxPre- and Posttest.docx [file mep_2374-8265.11540-s001.zip › C. Direct Observation Checklist.docx]

**Part 2: Direct Observation Checklist for Telemedicine Visits**

Overview

Direct observation is associated with positive educational outcomes and is recommended for telemedicine skill assessments. Consistent with direct observation best practices, this direct observation checklist is intentionally short, uses narrative descriptors rather than numerical scales, includes free-text responses to emphasize formative feedback, and instructs faculty to share feedback with residents immediately following the observation. While the direct observation is not overly prescriptive to increase the likelihood of participation, recommendations for implementation are included below for guidance.

Objectives

1. Perform direct observation of resident telemedicine skills in clinic to provide formative feedback.
2. Assess preceptor confidence in resident care delivered through telemedicine.

Direct Observation Set-Up

Ask each resident to identify 1-2 telemedicine visits, preferably video visits, on his or her schedule that would be opportunities for direct observation. The resident should communicate these opportunities with preceptors.

At our program, a continuity clinic visit typically has 3 parts: 1) A resident gathers history and performs the physical exam, 2) the resident leaves the patient encounter to precept with a faculty member, and 3) the resident returns to the patient to counsel about the plan that was discussed with the preceptor.

When a faculty performs a direct observation, he or she typically joins for part 1 and exits during part 2 or joins for part 3 after precepting with the resident. Consistent with this pattern, the checklist instructs the preceptor to observe either A) History and Physical OR B) counseling on the plan and to observe communication skills in either setting.

Please adapt the direct observation set-up to institutional clinic work-flows as needed.

Conducing Direct Observation

1. Complete the demographic information on the checklist form.
2. Discuss with the resident if you will observe **A) History and Physical OR B) Counseling on the Plan**.
3. Join the visit in-person or remotely.
   1. Accompany the resident to the exam room where the resident is doing the video visit.

OR

- 1. Join the video visit from your computer workstation. (For EPIC-EHR, click on the green camera icon.)

1. Ask the resident to introduce your role in the visit: “My preceptor (OR supervising doctor) is going to observe part of our visit today to give me feedback on my telemedicine skills. This is Dr. XXX.”
2. Observe the resident and complete the section for Communication Skills, either section A) or B), and Comments.
3. Provide 1-5min of feedback to the resident about telemedicine communication skills, completion of history and physical or counseling on the plan, and general observations.
4. Complete the last 2 items at the bottom of the checklist form and return it as recommended.

Direct Observation: Telemedicine Visit

Instructions for Preceptors: Please observe each resident perform a telemedicine visit, preferably a video visit, by attending either the **A) History and Physical** OR **B) Counseling on the Plan**. Complete the checklist below, providing observations on **Communication Skills** for each visit and provide real-time feedback to the resident after the visit.

Resident’s Name: Preceptor’s Name: Date:

Component(s) of visit observed: [ ] History/Physical [ ] Counseling on the Plan

| **Communication Skills:** complete with section A or B | Performed Well | Needs Improvement | Did Not Observe |
| --- | --- | --- | --- |
| Use of effective verbal communication skills |  |  |  |
| Use of effective non-verbal communication skills |  |  |  |
| Ability to overcome communication “stuck points” |  |  |  |
| Effective time management during visit |  |  |  |

| 1. **History and Physical** | Performed Well | Needs Improvement | Did Not Observe |
| --- | --- | --- | --- |
| Setting of agenda for visit |  |  |  |
| Ability to obtain a thorough and relevant history |  |  |  |
| Ability to perform appropriate physical exam given patient’s complaints and within constraints of video encounter |  |  |  |

| 1. **Counseling on the Plan** | Performed Well | Needs Improvement | Did Not Observe |
| --- | --- | --- | --- |
| Use of effective communication strategies to counsel on the plan (ex. teach back, avoid jargon) |  |  |  |
| Ability to explain next to steps to obtain labs or complete referrals |  |  |  |
| Communication of plan for follow-up appointment |  |  |  |

**Comments:**

| **What did the resident do well?** | **What can the resident improve?** |
| --- | --- |
|  |  |

Did you provide resident with feedback after the observed visit? [ ] Yes [ ] No

How confident are you in the quality of care delivered by the resident during the observed video visit?

| [ ] Not confident | [ ] Slightly confident | [ ] Moderately confident | [ ] Very confident |
| --- | --- | --- | --- |
