# Supplementary material for: Training Internal Medicine Residents to Perform Telemedicine Visits: A Novel Skill-Based Curriculum
Source: MedEdPORTAL. 2025 Jul 8;21:11540. doi: 10.15766/mep_2374-8265.11540 (PMC12237798; doi:10.15766/mep_2374-8265.11540)
Supplement: Supplementary file 1 — Faculty Facilitator Guide.docxResident Handout.docxDirect Observation Checklist.docxTelehealth Faculty Development Session.pptxPre- and Posttest.docx [file mep_2374-8265.11540-s001.zip › D. Telehealth Faculty Development Session.pptx]

## Slide 1
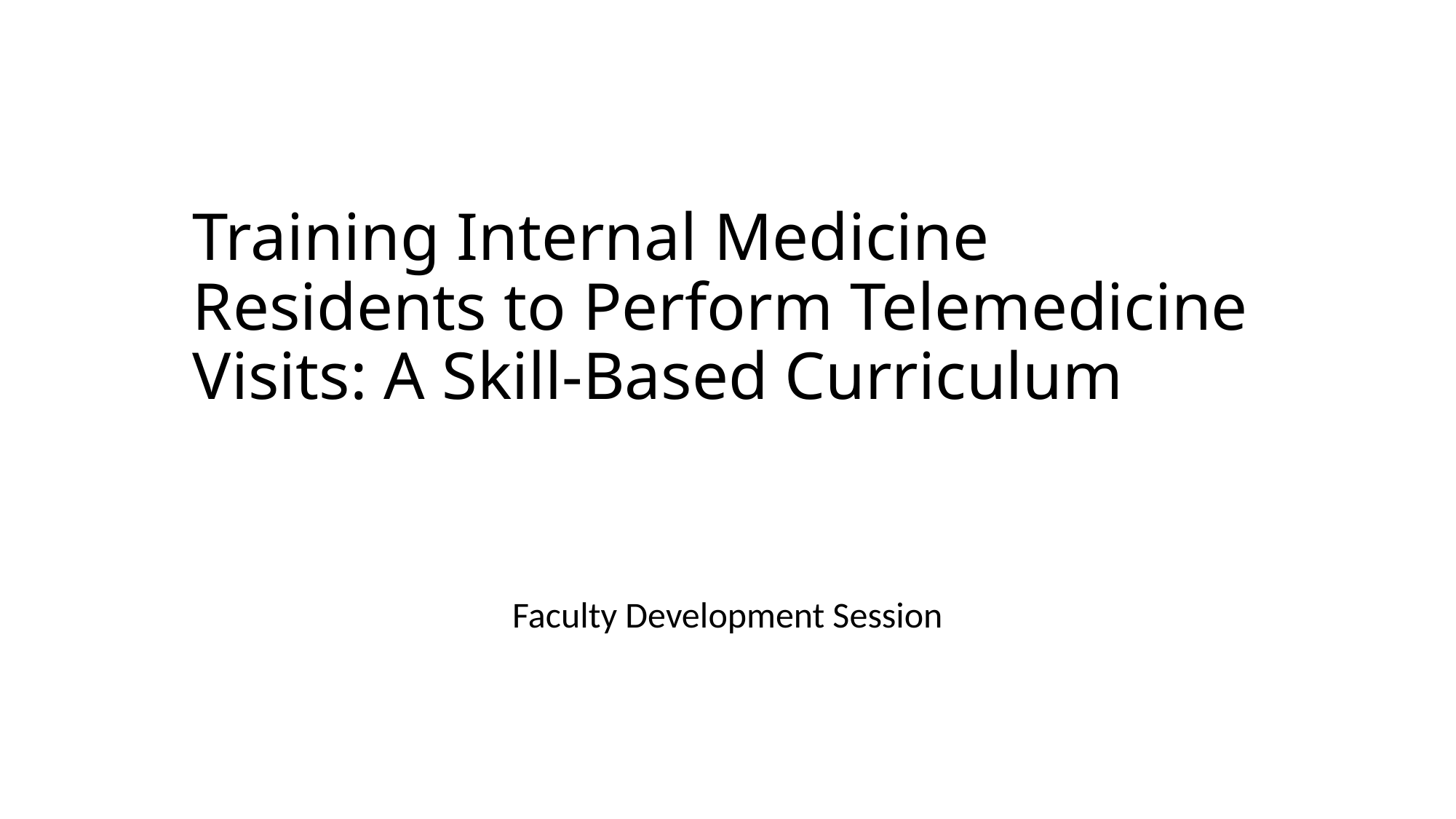

# Training Internal Medicine Residents to Perform Telemedicine Visits: A Skill-Based Curriculum
Faculty Development Session

## Slide 2
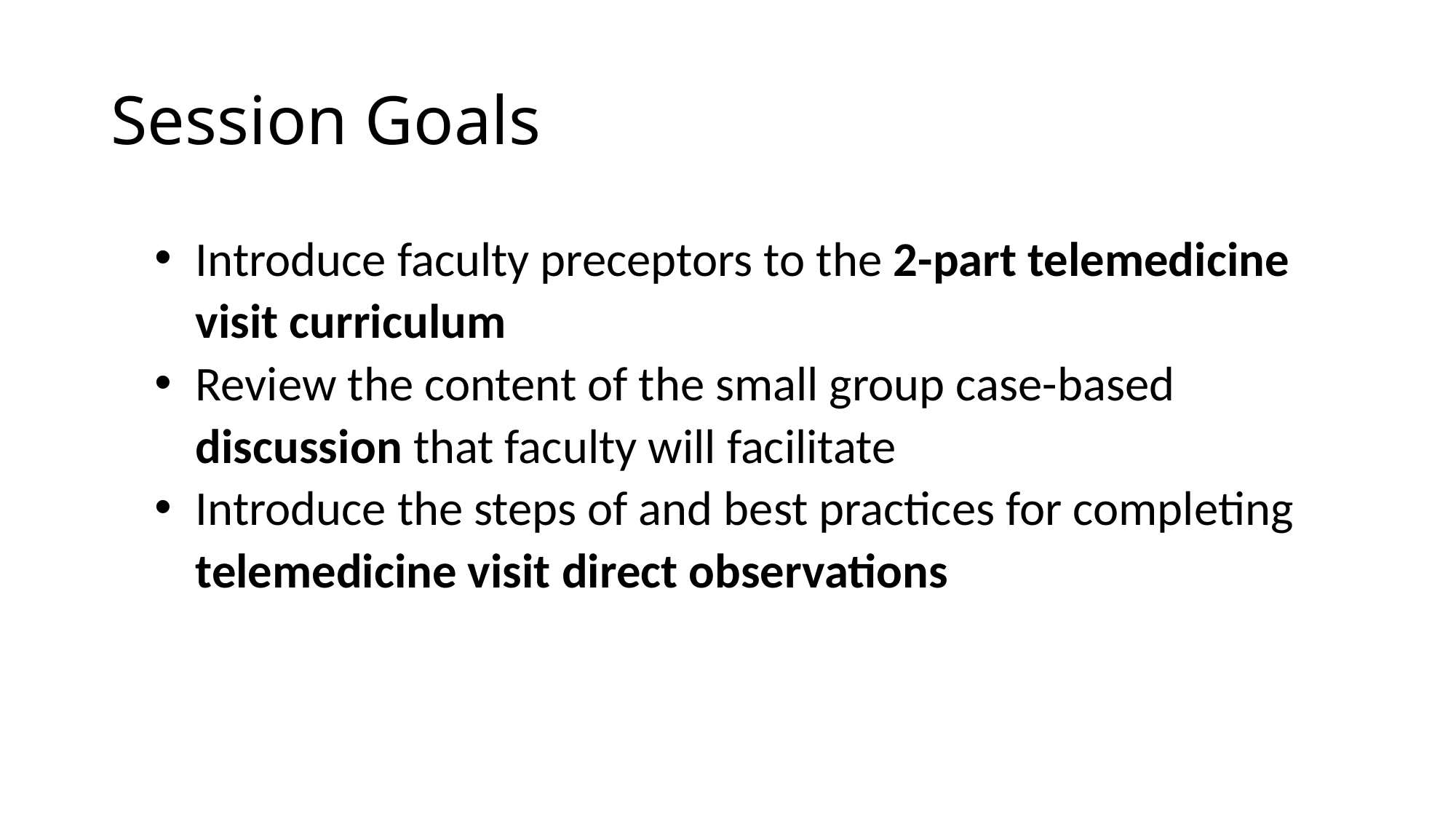

# Session Goals
Introduce faculty preceptors to the 2-part telemedicine visit curriculum
Review the content of the small group case-based discussion that faculty will facilitate
Introduce the steps of and best practices for completing telemedicine visit direct observations

## Slide 3
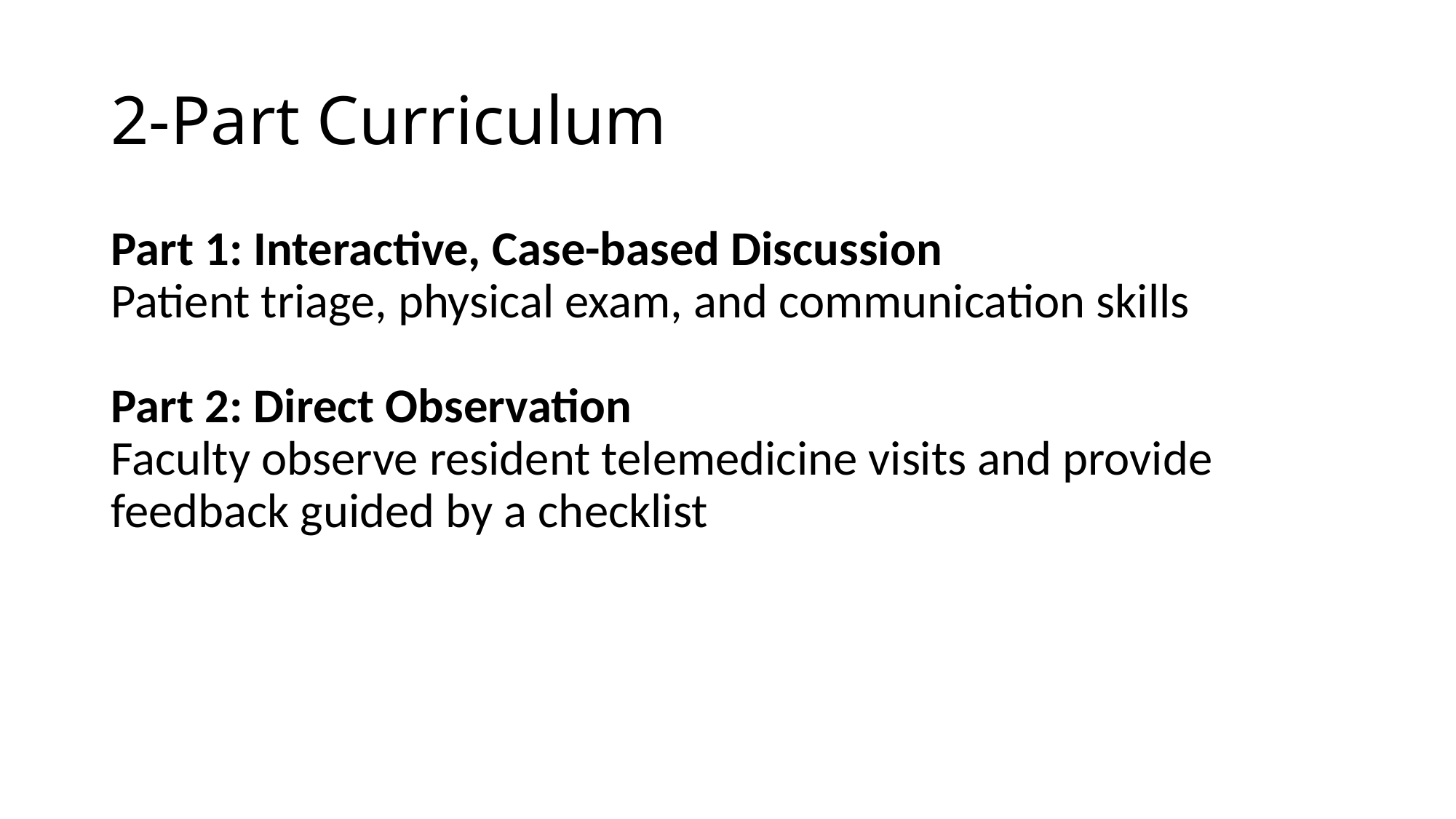

# 2-Part Curriculum
Part 1: Interactive, Case-based Discussion
Patient triage, physical exam, and communication skills
Part 2: Direct Observation
Faculty observe resident telemedicine visits and provide feedback guided by a checklist

## Slide 4
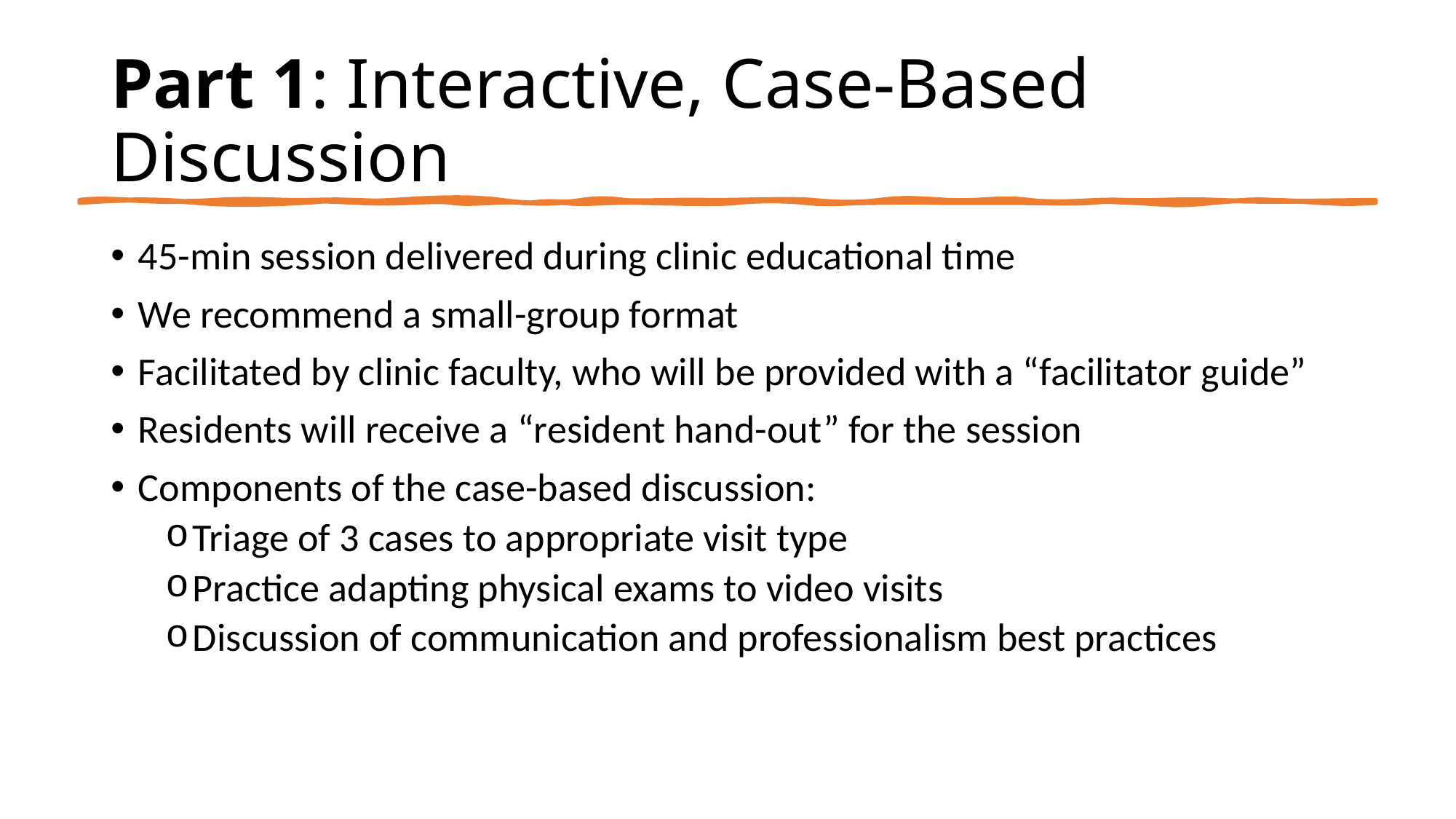

# Part 1: Interactive, Case-Based Discussion
45-min session delivered during clinic educational time
We recommend a small-group format
Facilitated by clinic faculty, who will be provided with a “facilitator guide”
Residents will receive a “resident hand-out” for the session
Components of the case-based discussion:
Triage of 3 cases to appropriate visit type
Practice adapting physical exams to video visits
Discussion of communication and professionalism best practices

## Slide 5
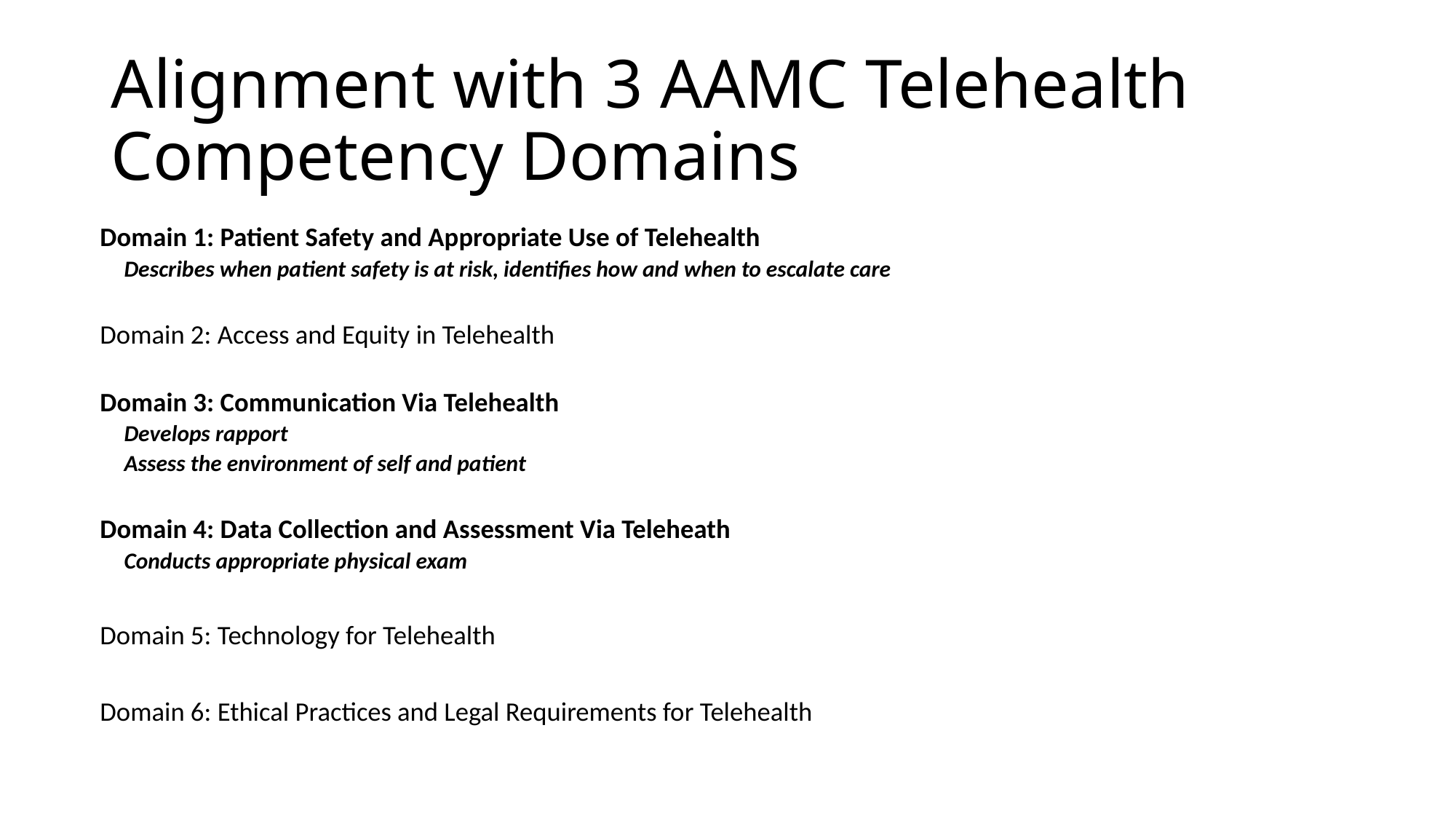

# Alignment with 3 AAMC Telehealth Competency Domains
Domain 1: Patient Safety and Appropriate Use of Telehealth
Describes when patient safety is at risk, identifies how and when to escalate care
Domain 2: Access and Equity in Telehealth
Domain 3: Communication Via Telehealth
Develops rapport
Assess the environment of self and patient
Domain 4: Data Collection and Assessment Via Teleheath
Conducts appropriate physical exam
Domain 5: Technology for Telehealth
Domain 6: Ethical Practices and Legal Requirements for Telehealth
AAMC Telehealth Competencies. Pre-publication Copy. Sept 2020.

## Slide 6
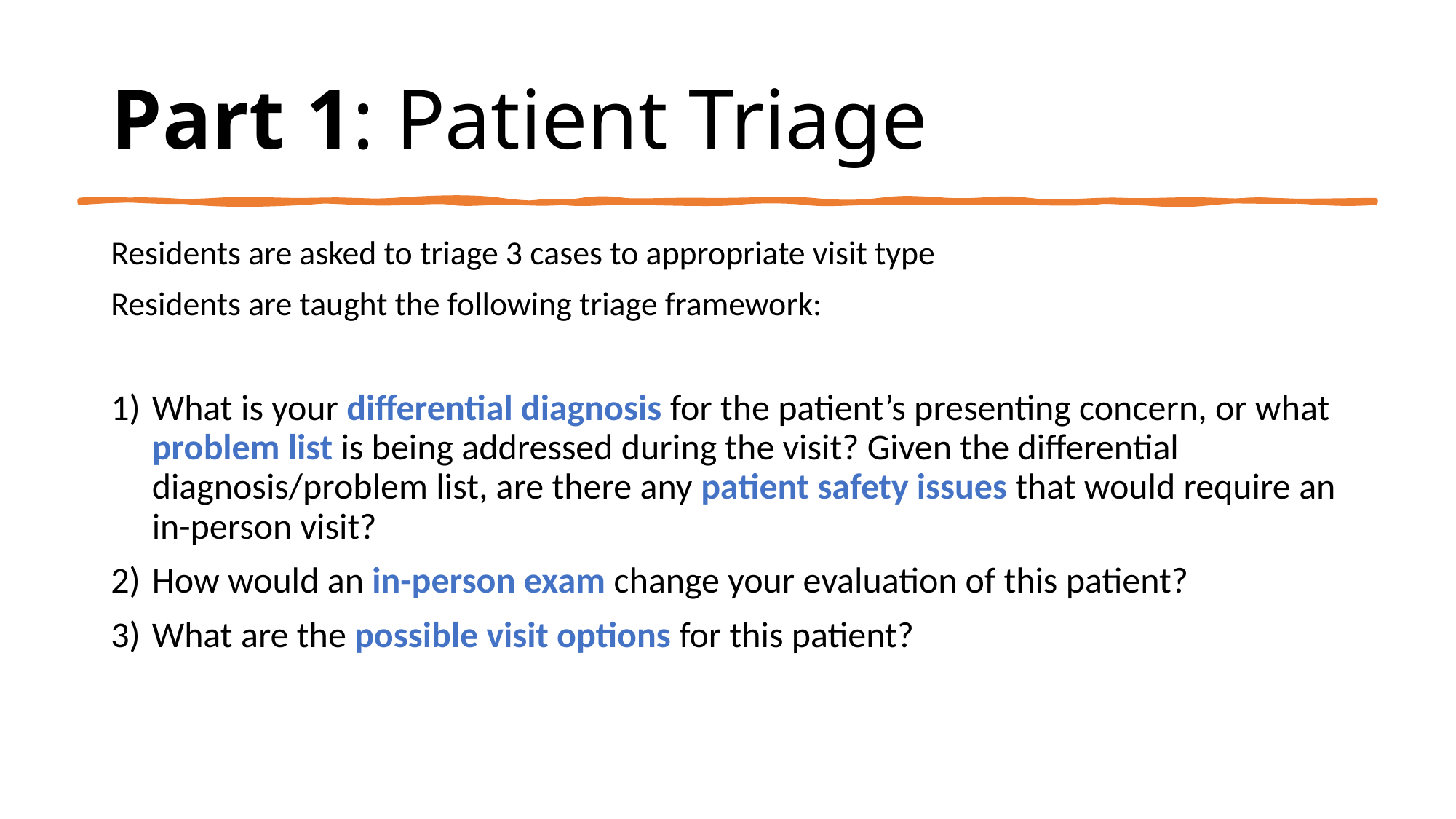

# Part 1: Patient Triage
Residents are asked to triage 3 cases to appropriate visit type
Residents are taught the following triage framework:
What is your differential diagnosis for the patient’s presenting concern, or what problem list is being addressed during the visit? Given the differential diagnosis/problem list, are there any patient safety issues that would require an in-person visit?
How would an in-person exam change your evaluation of this patient?
What are the possible visit options for this patient?

## Slide 7
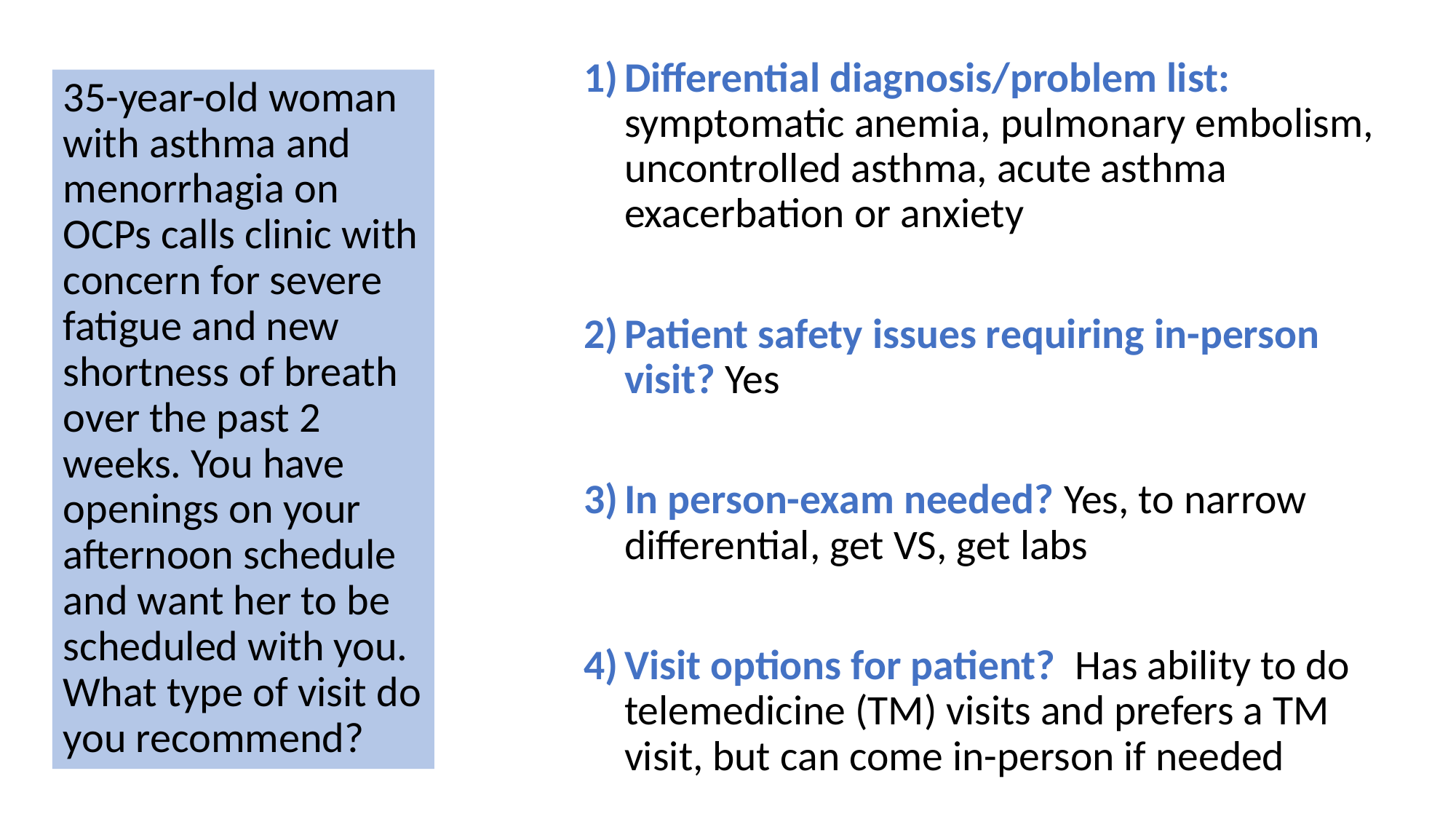

Differential diagnosis/problem list: symptomatic anemia, pulmonary embolism, uncontrolled asthma, acute asthma exacerbation or anxiety
Patient safety issues requiring in-person visit? Yes
In person-exam needed? Yes, to narrow differential, get VS, get labs
Visit options for patient? Has ability to do telemedicine (TM) visits and prefers a TM visit, but can come in-person if needed
35-year-old woman with asthma and menorrhagia on OCPs calls clinic with concern for severe fatigue and new shortness of breath over the past 2 weeks. You have openings on your afternoon schedule and want her to be scheduled with you. What type of visit do you recommend?

## Slide 8
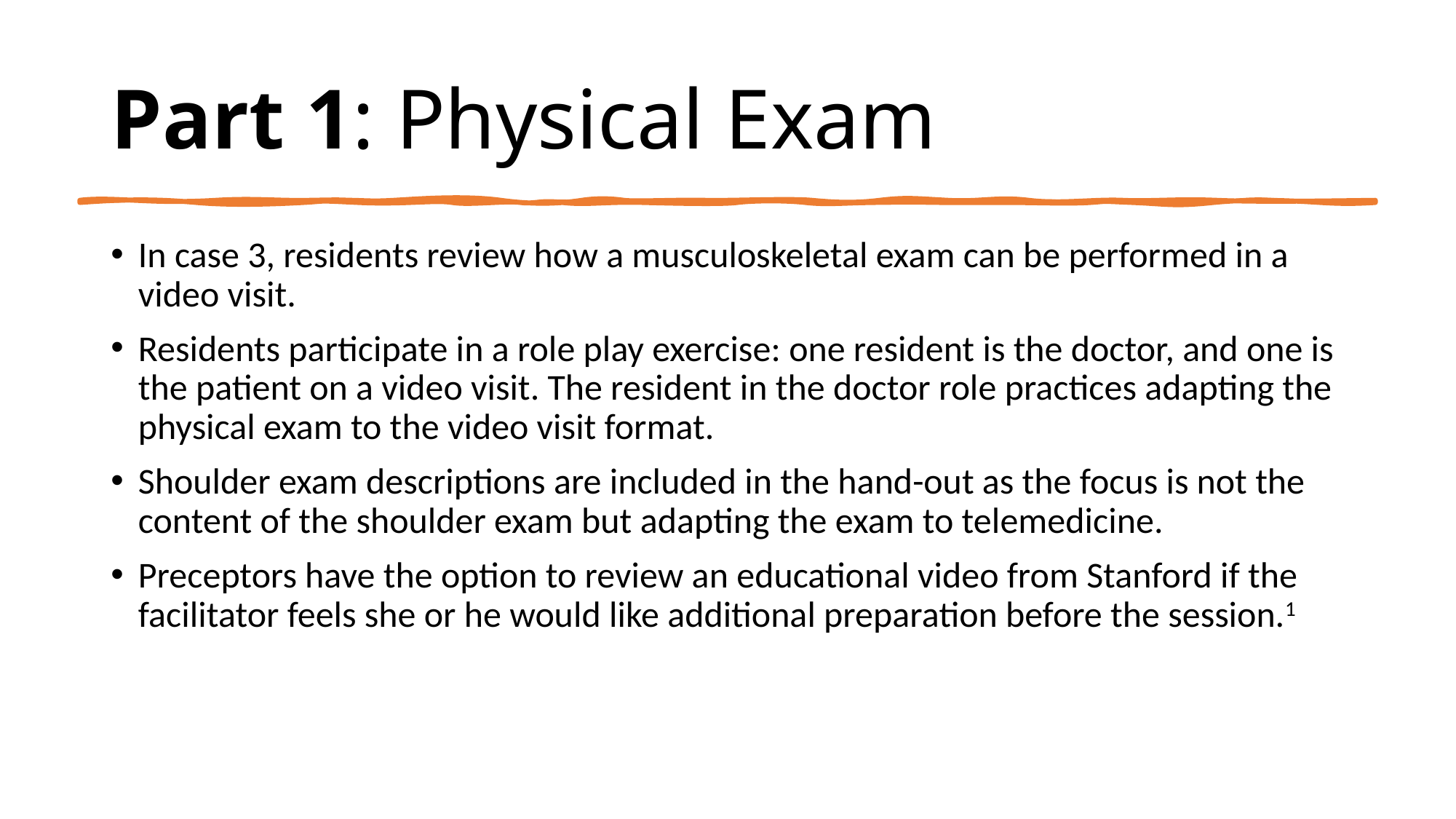

# Part 1: Physical Exam
In case 3, residents review how a musculoskeletal exam can be performed in a video visit.
Residents participate in a role play exercise: one resident is the doctor, and one is the patient on a video visit. The resident in the doctor role practices adapting the physical exam to the video visit format.
Shoulder exam descriptions are included in the hand-out as the focus is not the content of the shoulder exam but adapting the exam to telemedicine.
Preceptors have the option to review an educational video from Stanford if the facilitator feels she or he would like additional preparation before the session.1

## Slide 9
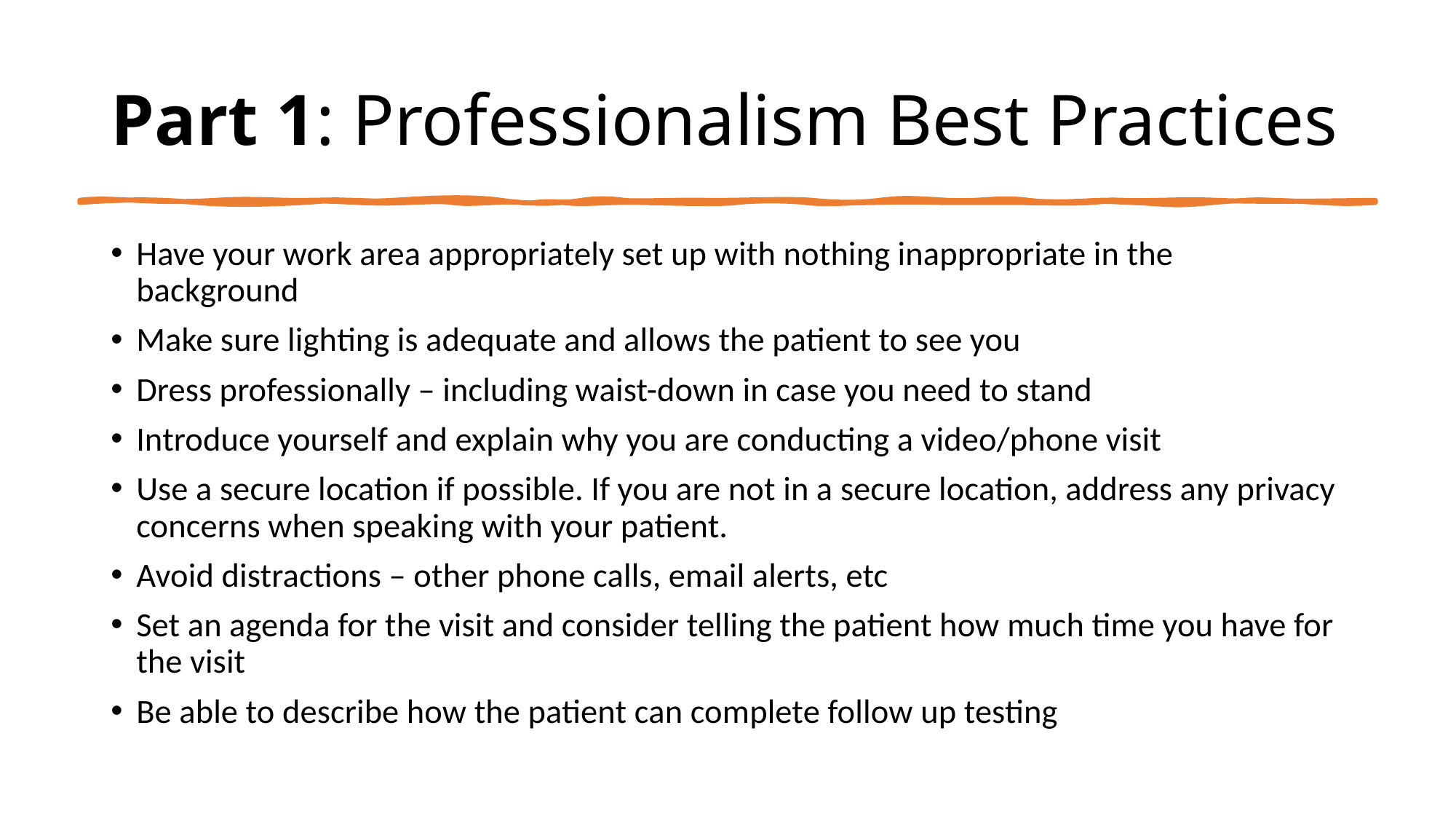

# Part 1: Professionalism Best Practices
Have your work area appropriately set up with nothing inappropriate in the background
Make sure lighting is adequate and allows the patient to see you
Dress professionally – including waist-down in case you need to stand
Introduce yourself and explain why you are conducting a video/phone visit
Use a secure location if possible. If you are not in a secure location, address any privacy concerns when speaking with your patient.
Avoid distractions – other phone calls, email alerts, etc
Set an agenda for the visit and consider telling the patient how much time you have for the visit
Be able to describe how the patient can complete follow up testing

## Slide 10
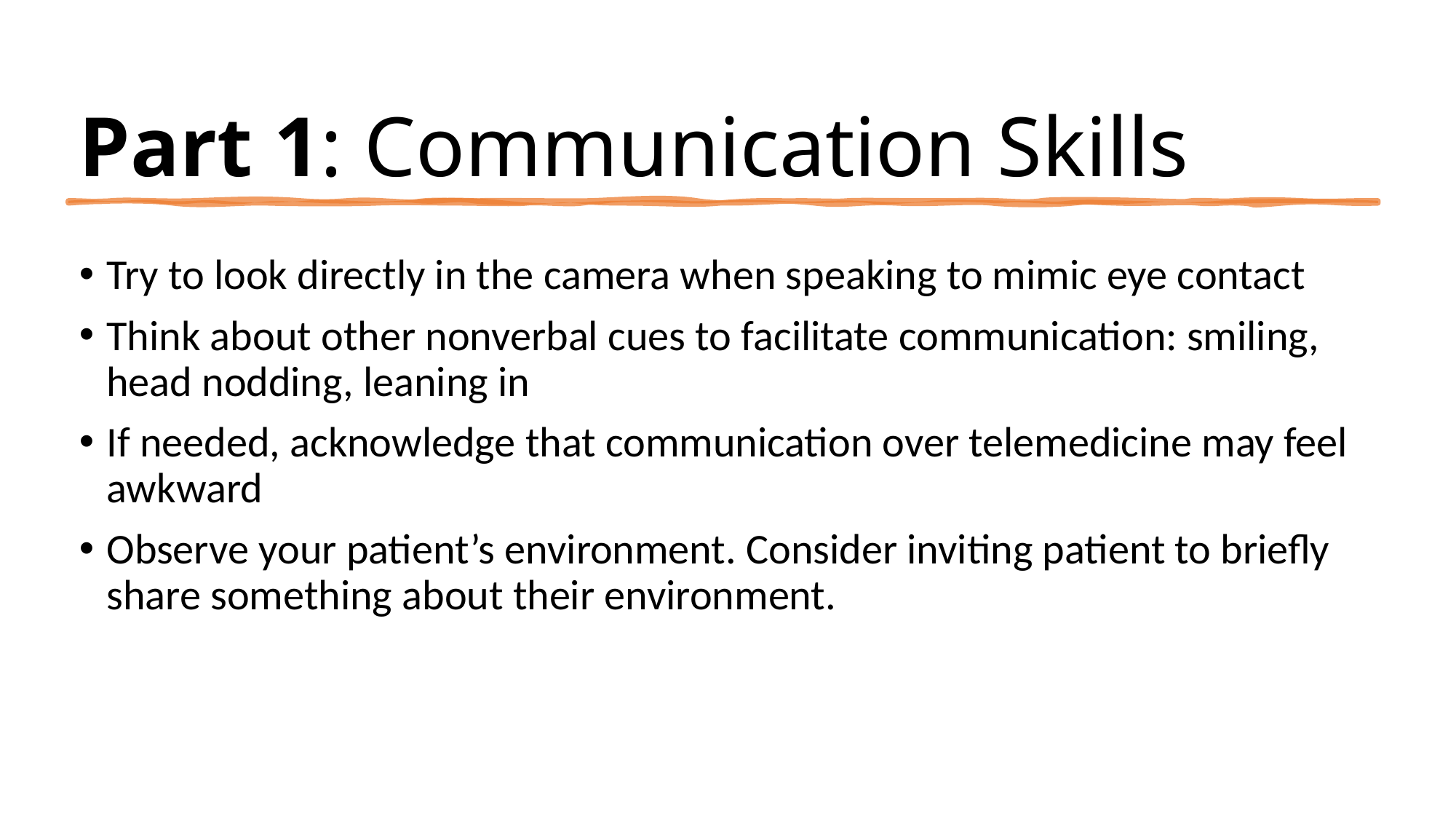

# Part 1: Communication Skills
Try to look directly in the camera when speaking to mimic eye contact
Think about other nonverbal cues to facilitate communication: smiling, head nodding, leaning in
If needed, acknowledge that communication over telemedicine may feel awkward
Observe your patient’s environment. Consider inviting patient to briefly share something about their environment.

## Slide 11
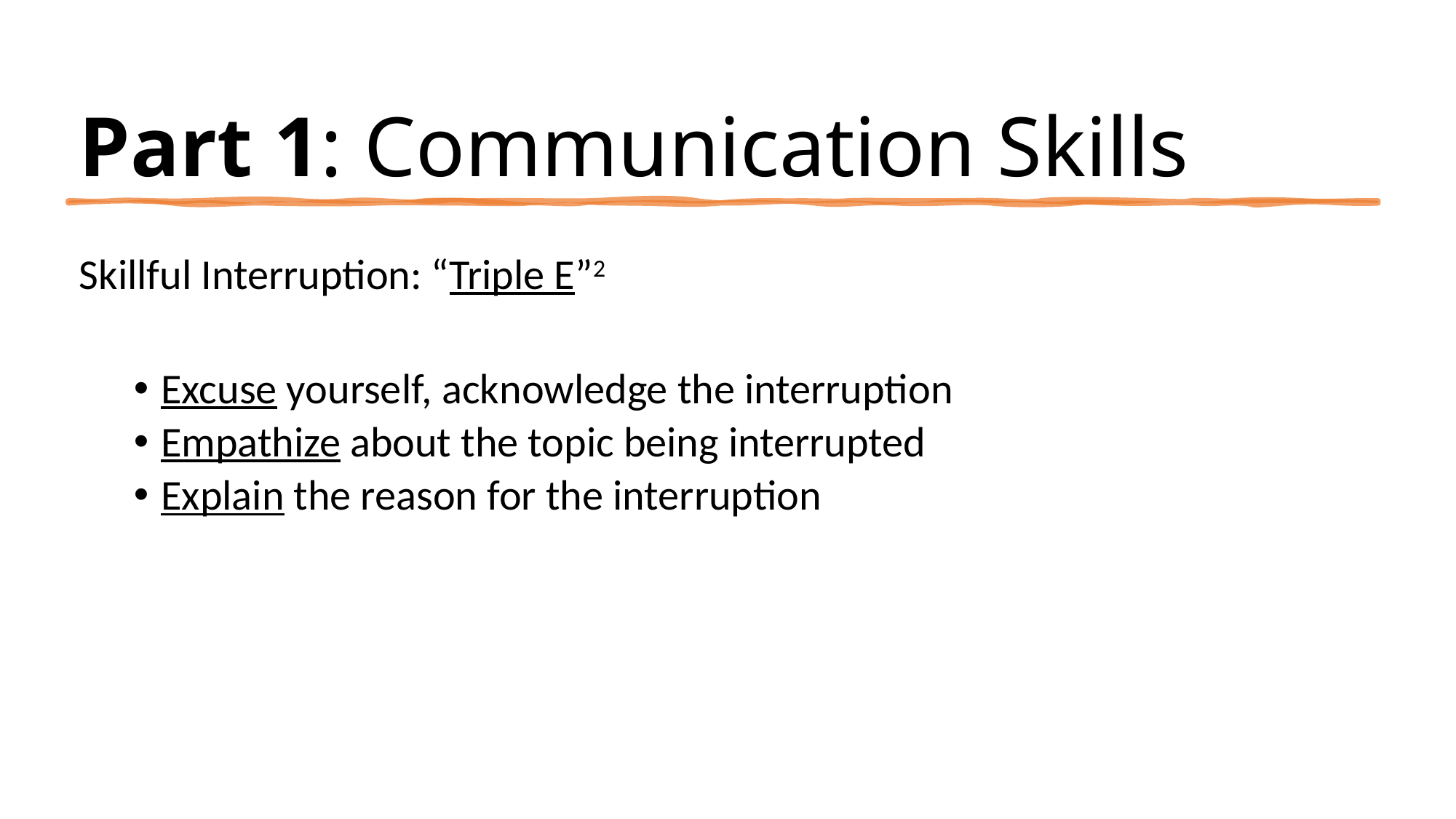

# Part 1: Communication Skills
Skillful Interruption: “Triple E”2
Excuse yourself, acknowledge the interruption
Empathize about the topic being interrupted
Explain the reason for the interruption

## Slide 12
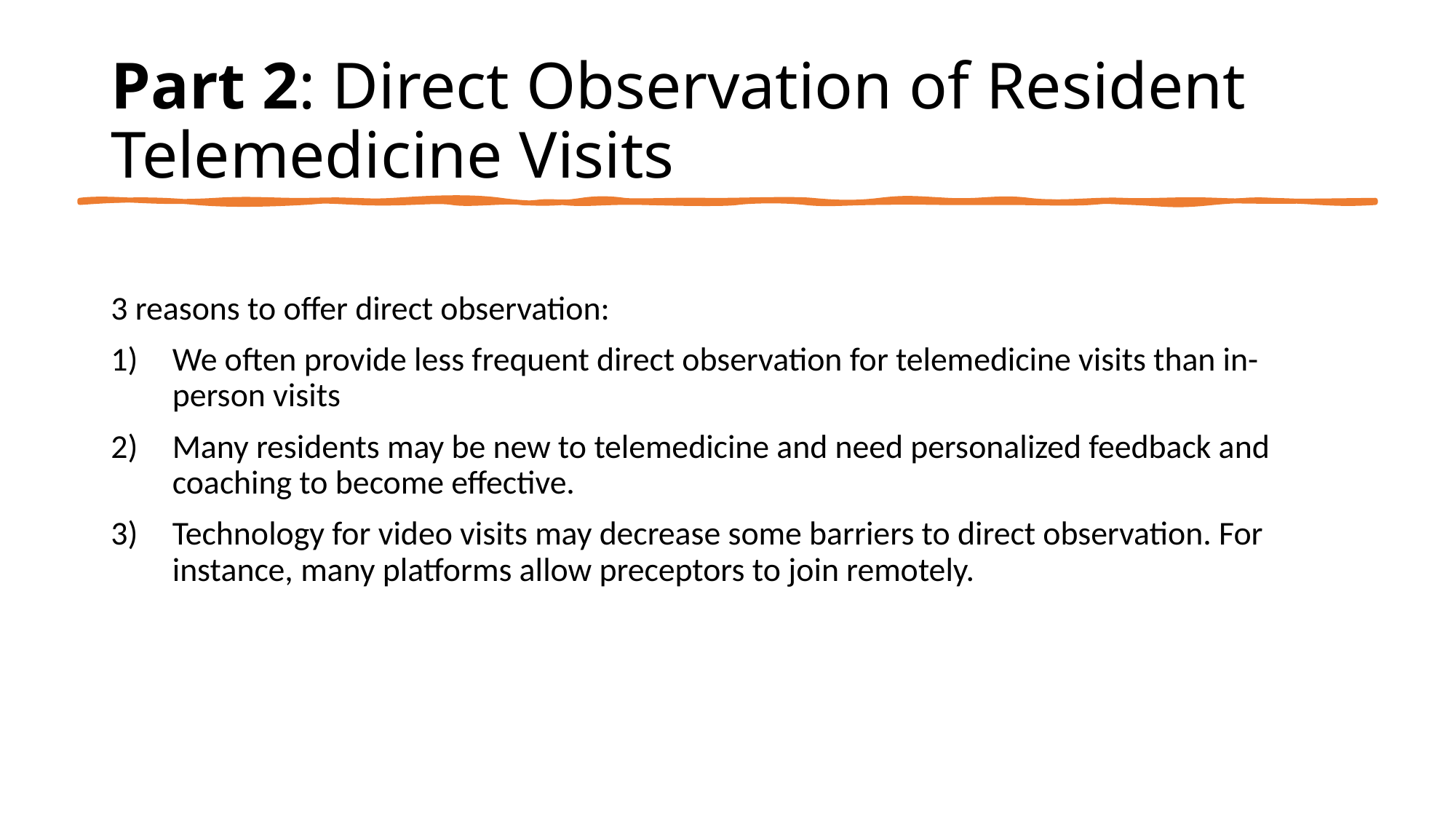

# Part 2: Direct Observation of Resident Telemedicine Visits
3 reasons to offer direct observation:
We often provide less frequent direct observation for telemedicine visits than in-person visits
Many residents may be new to telemedicine and need personalized feedback and coaching to become effective.
Technology for video visits may decrease some barriers to direct observation. For instance, many platforms allow preceptors to join remotely.

## Slide 13
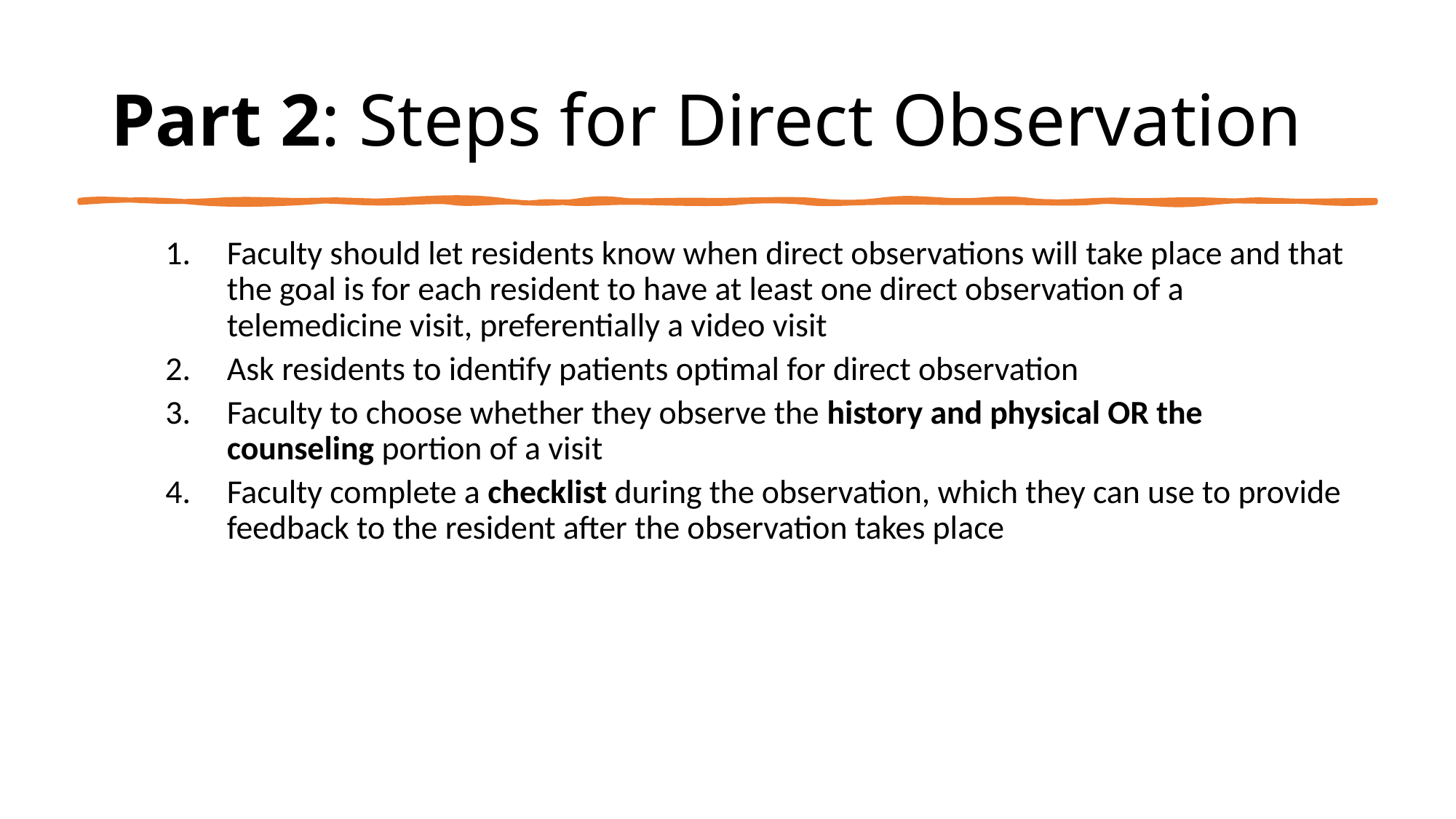

# Part 2: Steps for Direct Observation
Faculty should let residents know when direct observations will take place and that the goal is for each resident to have at least one direct observation of a telemedicine visit, preferentially a video visit
Ask residents to identify patients optimal for direct observation
Faculty to choose whether they observe the history and physical OR the counseling portion of a visit
Faculty complete a checklist during the observation, which they can use to provide feedback to the resident after the observation takes place

## Slide 14
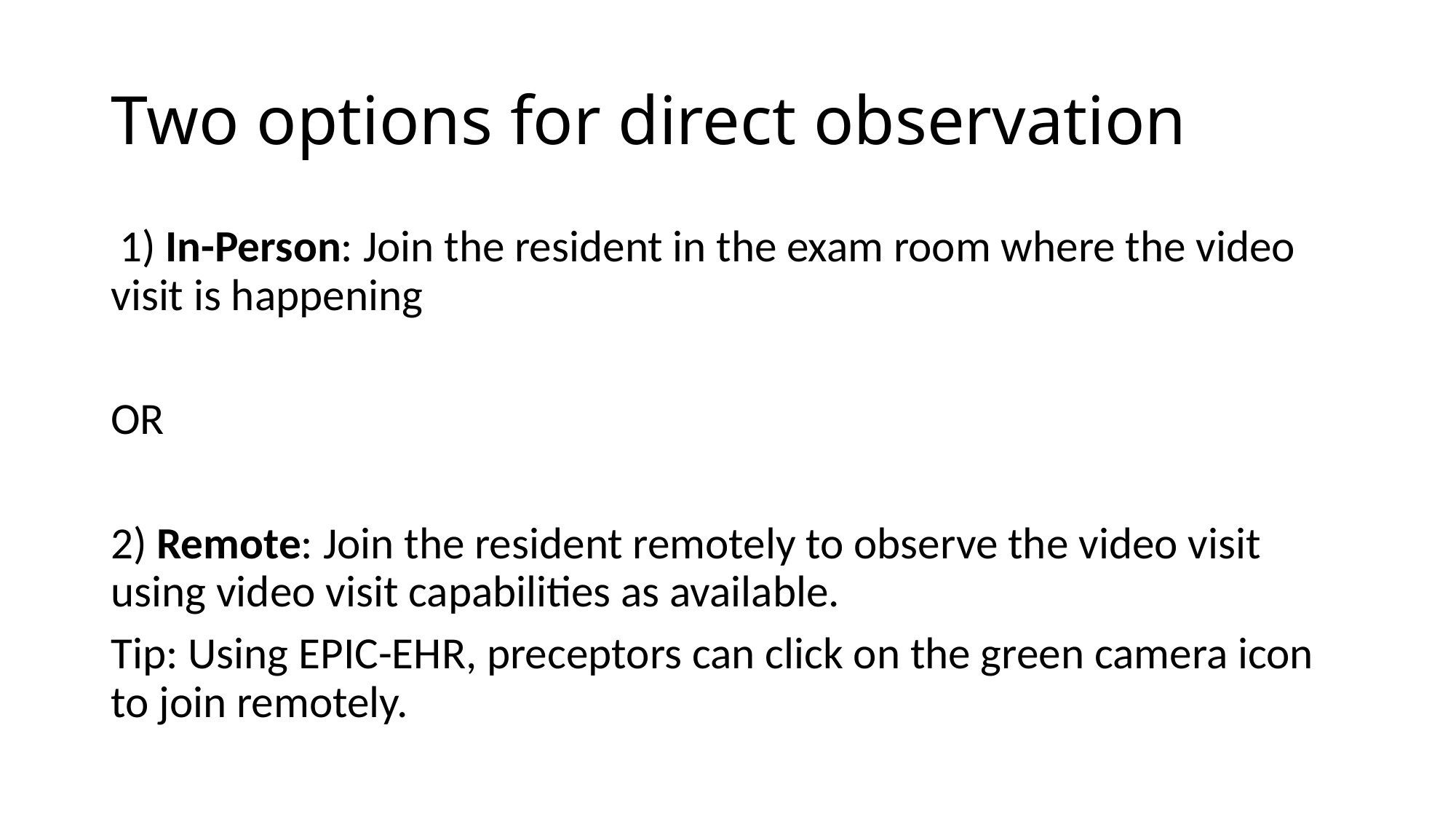

# Two options for direct observation
 1) In-Person: Join the resident in the exam room where the video visit is happening
OR
2) Remote: Join the resident remotely to observe the video visit using video visit capabilities as available.
Tip: Using EPIC-EHR, preceptors can click on the green camera icon to join remotely.

## Slide 15
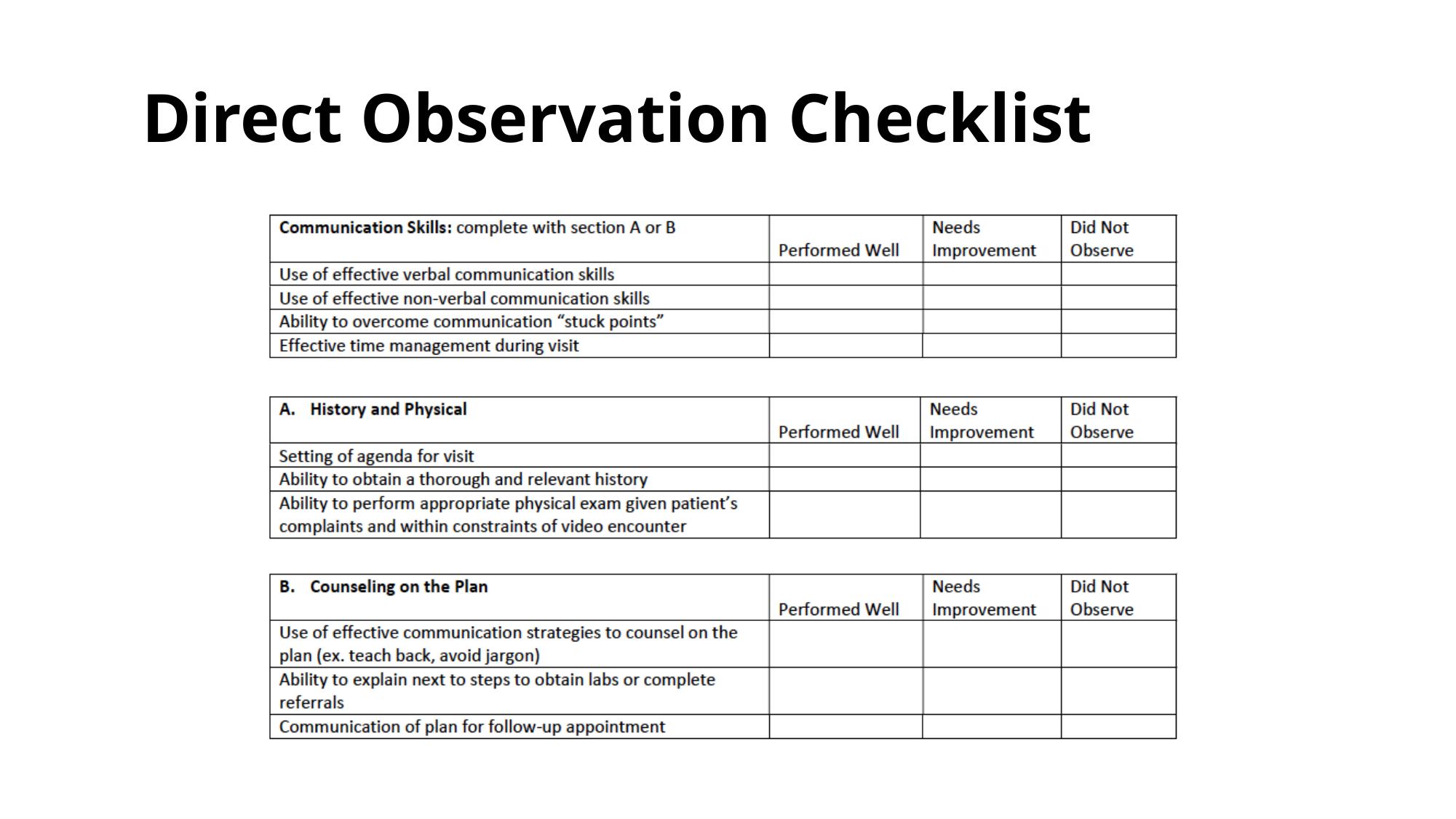

# Direct Observation Checklist

## Slide 16
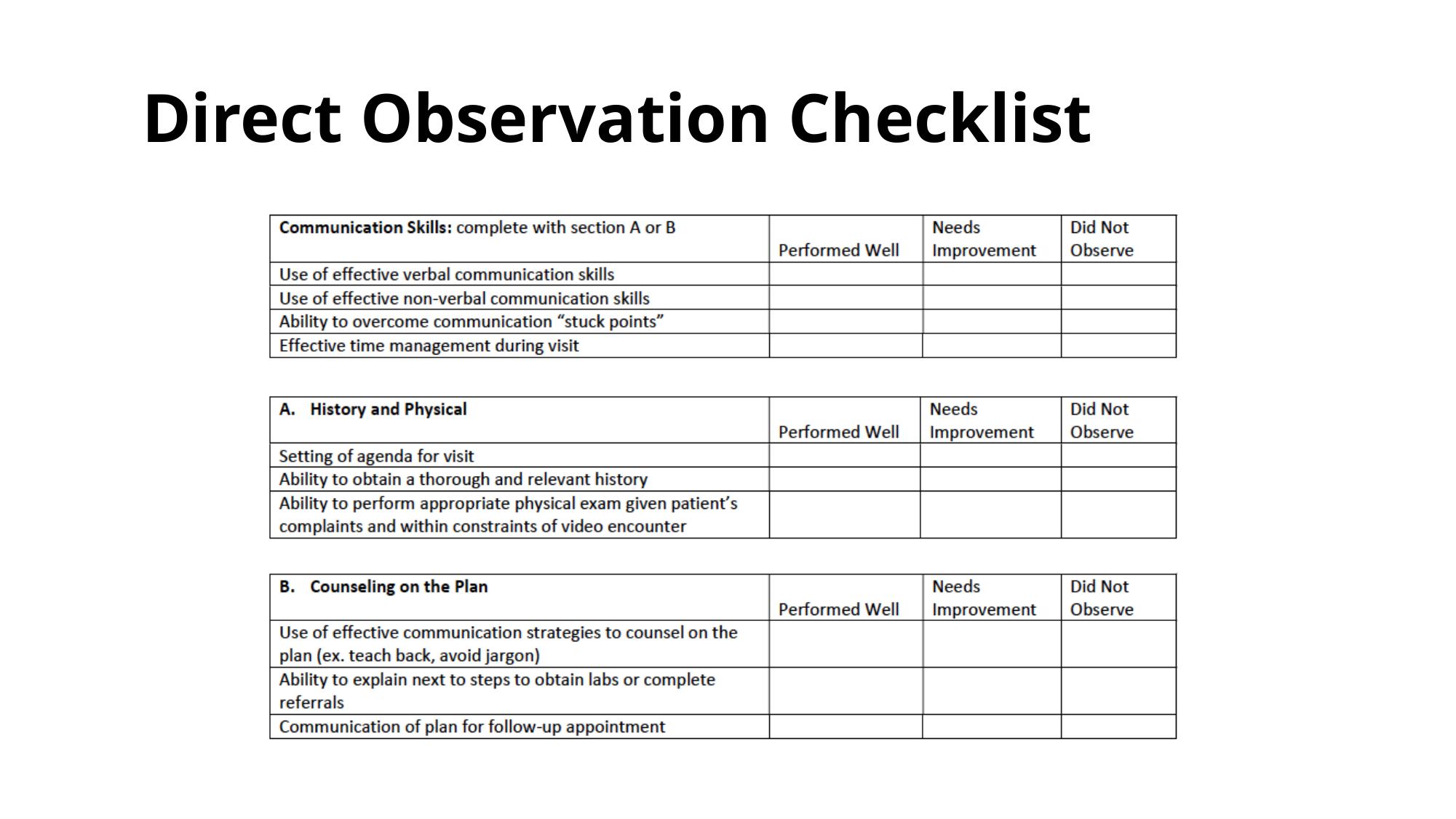

# Direct Observation Checklist

## Slide 17
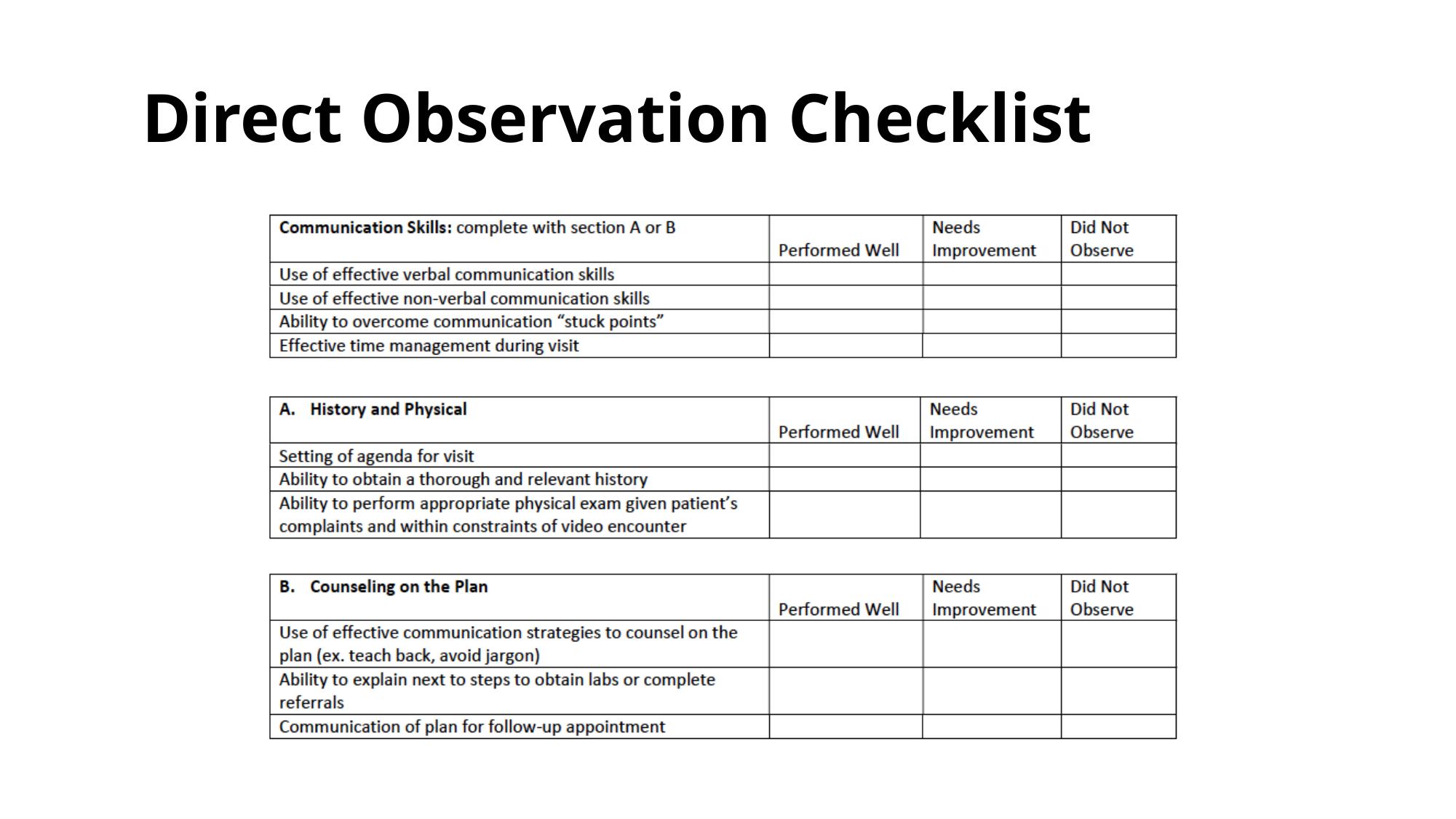

# Direct Observation Checklist

## Slide 18
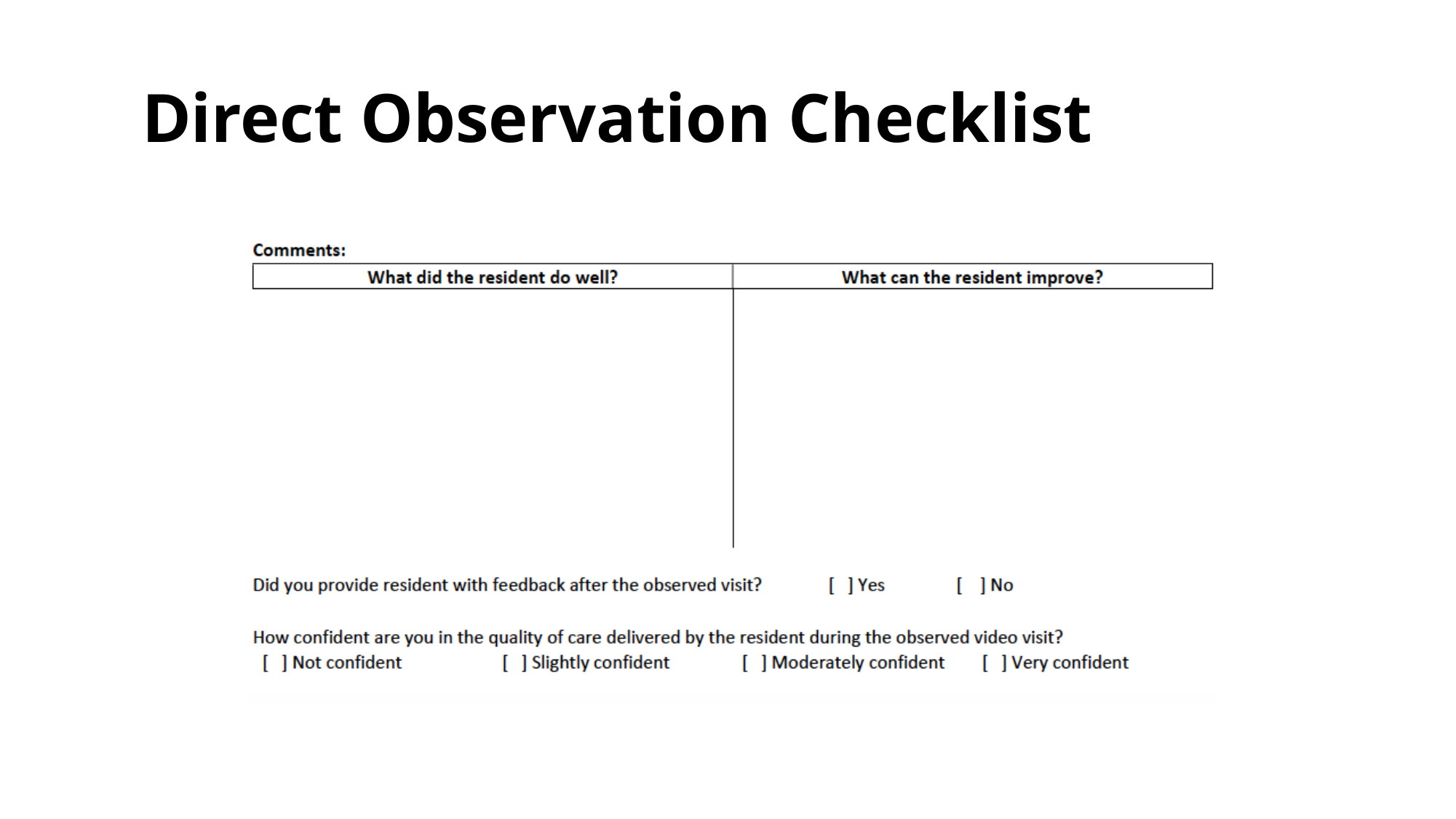

# Direct Observation Checklist

## Slide 19
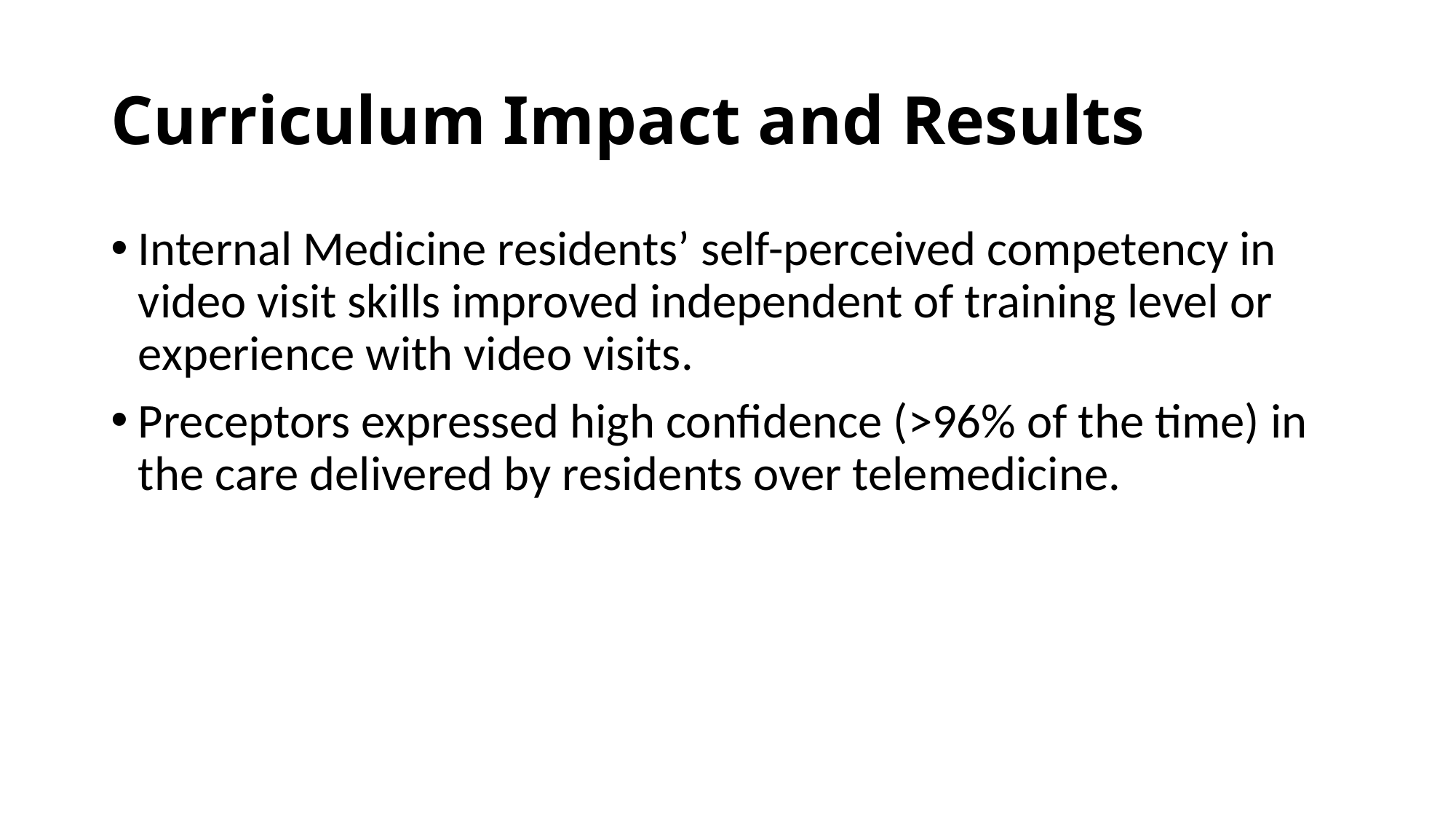

# Curriculum Impact and Results
Internal Medicine residents’ self-perceived competency in video visit skills improved independent of training level or experience with video visits.
Preceptors expressed high confidence (>96% of the time) in the care delivered by residents over telemedicine.

## Slide 20
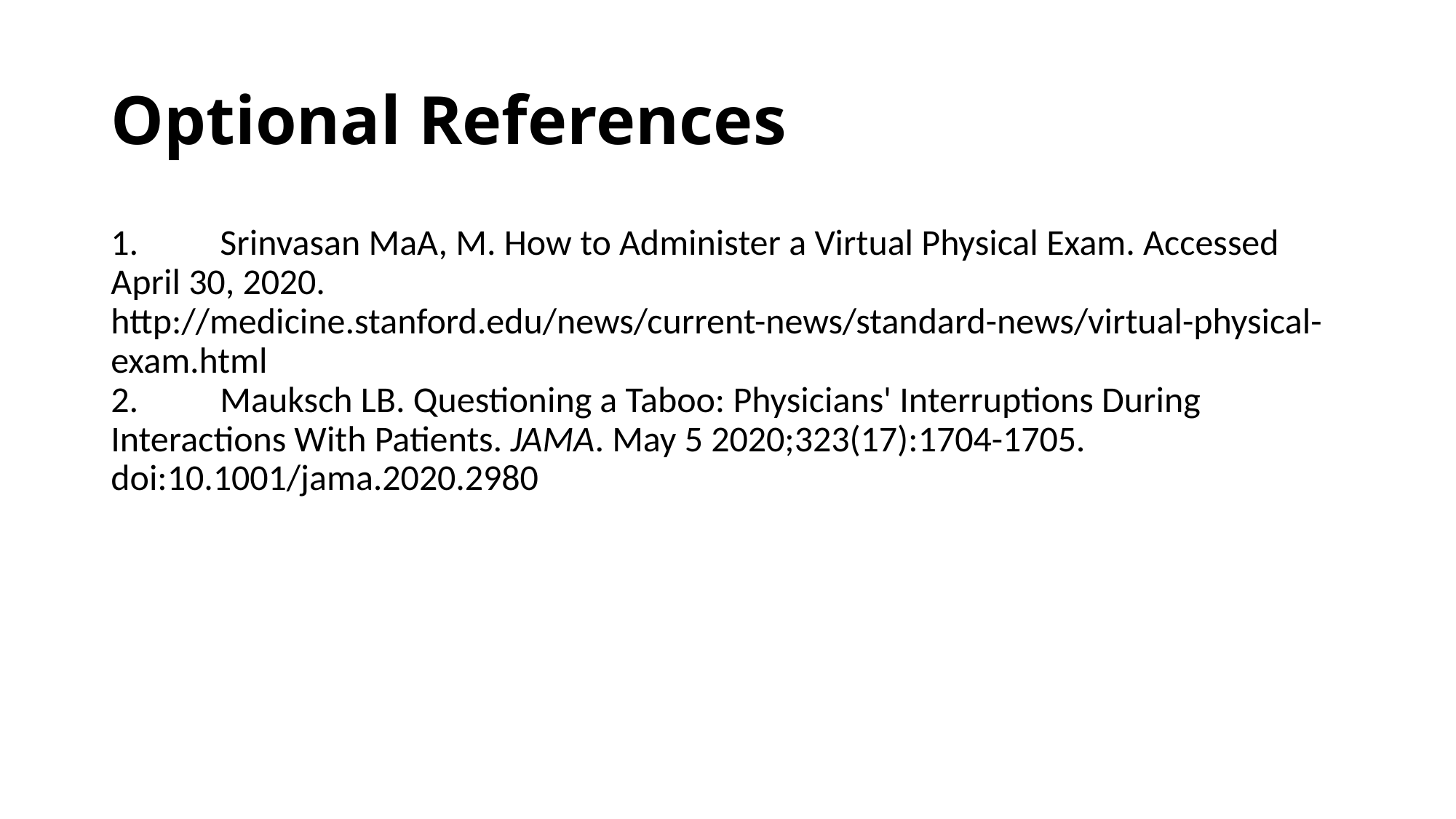

# Optional References
1.	Srinvasan MaA, M. How to Administer a Virtual Physical Exam. Accessed April 30, 2020. http://medicine.stanford.edu/news/current-news/standard-news/virtual-physical-exam.html
2.	Mauksch LB. Questioning a Taboo: Physicians' Interruptions During Interactions With Patients. JAMA. May 5 2020;323(17):1704-1705. doi:10.1001/jama.2020.2980
