# Supplementary material for: Training Internal Medicine Residents to Perform Telemedicine Visits: A Novel Skill-Based Curriculum
Source: MedEdPORTAL. 2025 Jul 8;21:11540. doi: 10.15766/mep_2374-8265.11540 (PMC12237798; doi:10.15766/mep_2374-8265.11540)
Supplement: Supplementary file 1 — Faculty Facilitator Guide.docxResident Handout.docxDirect Observation Checklist.docxTelehealth Faculty Development Session.pptxPre- and Posttest.docx [file mep_2374-8265.11540-s001.zip › E. Pre- and Posttest.docx]

The following survey questions apply to your experience with telemedicine video and phone encounters in your resident continuity clinic only.

How would you rate your efficiency with telemedicine **video** visits compared with in-person visits?

Much more efficient than with in-person visits

Somewhat more efficient than with in-person visits

Same degree of efficiency as with in-person visits

Somewhat less efficient than with in-person visits

Much less efficient than with in-person visits

How would you rate your efficiency with telemedicine **phone** visits compared with in-person visits?

Much more efficient than with in-person visits

Somewhat more efficient than with in-person visits

Same degree of efficiency as with in-person visits

Somewhat less efficient than with in-person visits

Much less efficient than with in-person visits

Attitudes towards telemedicine:

| Please rate your level of agreement with the following statements: | Strongly disagree | Somewhat disagree | Neither agree nor disagree | Somewhat agree | Strongly agree |
| --- | --- | --- | --- | --- | --- |
| Telemedicine has utility for managing patients’ acute concerns |  |  |  |  |  |
| Telemedicine has utility for managing patients’ chronic conditions |  |  |  |  |  |
| The quality of telemedicine visits is the same as in-person visits |  |  |  |  |  |
| I would like to use telemedicine in my future practice |  |  |  |  |  |

What is your level of competence in performing the following?

|  | Not competent | Slightly competent | Moderately competent | Very competent | Highly competent |  |
| --- | --- | --- | --- | --- | --- | --- |
| Determining when a patient concern is appropriate for a telemedicine visit versus in person visit |  |  |  |  |  | C1 |
| Managing patients’ **acute** concerns during telemedicine visits |  |  |  |  |  | C2 |
| Managing patients’ **chronic** conditions during telemedicine visits |  |  |  |  |  | C3 |
| Determining plan to address patients’ preventative care needs during telemedicine visits |  |  |  |  |  | C4 |

What is your level of competence in performing the following components of a telemedicine visit?

|  | Not competent | Slightly competent | Moderately competent | Very competent | Highly competent |  |
| --- | --- | --- | --- | --- | --- | --- |
| Using technology to connect with a patient over audio or video for a telemedicine visit |  |  |  |  |  | C5 |
| Collecting history through telemedicine |  |  |  |  |  | C6 |
| Performing physical exam through telemedicine |  |  |  |  |  | C7 |
| Counseling patients through telemedicine on the plan |  |  |  |  |  | C8 |
| Using the electronic health record during telemedicine visits (includes placing orders, writing notes, billing) |  |  |  |  |  | C9 |
| Presenting a patient evaluated through telemedicine to your clinic preceptor |  |  |  |  |  | C10 |
| Arranging for patient follow-up testing after a telemedicine visit |  |  |  |  |  | C11 |

What is your level of competence with the following **communication** skills?

|  | Not competent | Slightly competent | Moderately competent | Very competent | Highly competent |  |
| --- | --- | --- | --- | --- | --- | --- |
| Setting an agenda at the beginning of a telemedicine visit |  |  |  |  |  | C12 |
| Establishing an effective rapport with a patient during a telemedicine visit |  |  |  |  |  | C13 |
| Redirecting the conversation with a patient if needed during a telemedicine visit |  |  |  |  |  | C14 |
| Effective time management during a telemedicine visit |  |  |  |  |  | C15 |

**Demographics/Prior Training**

Prior to March 2020 when telemedicine was incorporated in continuity clinic as a result of the COVID-19 pandemic, did you have any **training** in how to conduct telemedicine visits?

Yes: (Please describe nature of training:_________)

No

Prior to March 2020 when telemedicine was incorporated in continuity clinic as a result of the COVID-19 pandemic, did you have any hands-on **experience** in conducting telemedicine visits?

Yes (Please describe nature of experience: _________)

No

What is your post-graduate year (PGY)?

PGY-1

PGY-2

PGY-3

PGY-4

At which clinic site(s) do you attend resident continuity clinic?

Please write-in:

What is your gender?

Male

Female

Prefer not to answer

Prefer to self-describe: ___
