# Supplementary material for: Training Internal Medicine Residents to Perform Telemedicine Visits: A Novel Skill-Based Curriculum
Source: MedEdPORTAL. 2025 Jul 8;21:11540. doi: 10.15766/mep_2374-8265.11540 (PMC12237798; doi:10.15766/mep_2374-8265.11540)
Supplement: Supplementary file 1 — Faculty Facilitator Guide.docxResident Handout.docxDirect Observation Checklist.docxTelehealth Faculty Development Session.pptxPre- and Posttest.docx [file mep_2374-8265.11540-s001.zip › _Educational Summary Report_11540.pdf]

MedEdPORTAL® The AAMC Journal of Teaching and Learning Resources

Original Publication Open Access

Training Internal Medicine Residents to Perform Telemedicine Visits: A Novel Skill-Based Curriculum

Sarah Jones, MD, MS*, Melissa McNeil, MD, MS, Scott D. Rothenberger, PhD, Kwonho Jeong, MS, Tanya Nikiforova, MD, MS

*Corresponding author: jonessa3@upmc.edu

Abstract

Introduction: Since the COVID-19 pandemic, internal medicine (IM) residents have provided patient care via telemedicine in their continuity clinics but often without formal training in telemedicine skills. To address this education gap, we developed a two-part curriculum to improve IM residents' self-perceived competence with outpatient telemedicine skills. **Methods:** From May to August 2020, IM residents participated in a 45-minute interactive case-based, small-group discussion focused on patient triage, virtual physical examination, and telemedicine communication skills. Faculty preceptors directly observed resident telemedicine visits and provided residents with formative feedback using a checklist. Residents completed pre- and postsession surveys assessing self-perceived competence with 15 telemedicine skills. **Results:** A total of 119 residents participated in the case-based session, and most (61%) received direct observation. Among these residents, 51% (61/119) completed both pre- and postsession surveys. After completing the curriculum, residents' self-perceived competence increased for all skills, with the largest gains in triaging patients to visit types, physical exam adaptation, addressing preventative care, and arranging follow-up (mean 0.5 increase in participant ratings based on 5-point Likert scale; $p < .001$). Improvement in self-perceived competence was independent of resident level of training and video visit volume. Faculty performing direct observations expressed high levels of confidence in the care delivered. **Discussion:** This easily implemented curriculum combining a case-based discussion with direct observation increased IM residents' self-perceived competence in telemedicine skills and could be readily adopted in IM residency programs and various specialties to provide trainees with telemedicine skills.

Keywords
Telemedicine, Telehealth, Direct Observation, Case-Based Learning, Internal Medicine

Educational Objectives

By the end of the activity, learners will be able to:

1. Identify clinical scenarios that are most appropriate for different visit types, including phone, video, and in person.
2. Perform a focused history and physical exam through a telemedicine visit.
3. Demonstrate essential skills necessary for efficient, timely telemedicine visits, including disease management, counseling, follow-up, technology use, and presenting to a clinical preceptor.
4. Name three best practices for effective communication during a telemedicine visit.

Citation:
Jones S, McNeil M, Rothenberger SD, Jeong K, Nikiforova T. Training internal medicine residents to perform telemedicine visits: a novel skill-based curriculum. *MedEdPORTAL*. 2025;21:11540.
https://doi.org/10.15766/mep_2374-8265.11540

Introduction

For years, outpatient telemedicine has provided successful management of chronic conditions and accurate diagnosis of patients' acute concerns.[1,2] With the COVID-19 pandemic, rapid adoption of telemedicine allowed for safe continuation of clinical care, and by April 2020, rates of telehealth care delivery had increased almost 10-fold in many health care systems.[3] Although telemedicine visit rates have since decreased, approximately 40% of adults have continued to utilize telemedicine visits, especially patients with more chronic medical conditions, more frequent visits, and transportation concerns.[4] Therefore, telemedicine remains an essential tool for internal medicine, and telemedicine skills can be learned during residency training.

Like many health care centers around the US, the University of Pittsburgh Medical Center rapidly responded to the pandemic with expanded telemedicine use; by April 2020, they were conducting half of all outpatient visits over video, which is similar to the national rates of peak pandemic telemedicine use.[3]

Copyright © 2025 Jones et al. This is an open-access publication distributed under the terms of the Creative Commons Attribution license.

Residents conducted telemedicine visits without standardized training, which was not atypical. Although almost 60% of medical schools were offering elective or required telemedicine curricula by 2018, the training was heterogeneous, ranging from broad introductory concepts to teleassessments.[5] Furthermore, telemedicine training was not a priority in graduate medical education, and the 2018 ACGME Milestones National Report of 104 specialties identified only one program, in child and adolescent psychiatry, that included telemedicine in their milestones.[5,6] Published telemedicine curricula in graduate medical education were difficult to identify prior to 2020, and at least one study reported that, unsurprisingly, residents have deficiencies in telemedicine skills.[7]

Recognizing and responding to the need for competencies in telemedicine care, the AAMC released draft telehealth competencies in late 2020, and the updated 2021 ACGME Milestones National Report (version 2.0) included telemedicine skills for internal medicine (IM) residents.[8,9] The AAMC defined telehealth competencies across six domains: (1) Patient Safety and Appropriate Use, (2) Access and Equity, (3) Communication, (4) Data Collection and Assessment, (5) Technology Use, and (6) Ethical Practices and Legal Requirements. Telemedicine education programs developed in response to pandemic-related telemedicine use generally preceded or occurred in parallel to development of these competencies.

Since 2020, structured telemedicine curricula have been published but most were designed for medical student learners rather than IM residents.[10-14] Only one telemedicine curriculum published on *MedEdPORTAL* was designed for residents, specifically within pediatric neurology.[15] Curricula for IM residents that have been published outside of *MedEdPORTAL* include an intensive and longitudinal 3-year program, a brief resident-led program focused on teaching workflow, communication, and physical examination by telemedicine, and a three-session curriculum focused on teaching legal guidelines, chronic disease management, health equity, and virtual physical examination.[16-18] Notably, although none of these curricula for IM residents focus on the important skill of patient triage by telemedicine, a curriculum for family medicine residents included patient triage by telemedicine.[19] Additionally, only one curriculum included a direct observation component for formative feedback.[17]

Telemedicine continues to serve an important role in health care delivery, and thus innovative curricula that can meet diverse program needs are beneficial, including curricula that are easily implemented within existing residency educational structures and require few additional resources. We developed an easy-to-implement skill-based telemedicine curriculum for IM residents, with a case-based discussion focused on patient triage, communication skills, and physical examination skills as well as formative direct observation. Broad goals for the curriculum were (1) to improve self-perceived competence with telemedicine skills, (2) to foster positive attitudes toward telemedicine, and (3) to increase direct observation and feedback on telemedicine skills in the clinic.

Methods

Educational Context

All 152 categorical IM residents (PGY 1, 2, and 3) at a large academic center in 2020 were eligible to participate in the curriculum. Residents participated in the curriculum either from May to June 2020 or from July to August 2020, depending on their clinic schedules (Figure). PGY 1 residents who started their postgraduate year in July 2020 were not eligible to participate in this curriculum as they had no experience with telemedicine at our institution, and therefore participated in a separate telemedicine orientation curriculum.

The Figure shows the real-time transition to delivering telemedicine care, including volume of video visits performed in EpicCare-based continuity clinics and the time line for the faculty needs assessment, curriculum delivery, and curriculum assessment, which will be discussed in more detail below.

Two-thirds of residents attended continuity clinics utilizing the EpicCare electronic health record (EHR) system with integrated video visit technology. Alternatively, residents used the Doximity platform if EpicCare-based technologies failed. Before this curriculum, residents at EpicCare-based clinics received informal instruction in telemedicine technology.

The other one-third of residents attended continuity clinics at the Veterans Administration (VA) health care system, where they utilized the Computerized Patient Record System EHR, and VA Video Connect, a VA-specific, secure video conferencing application. Residents at the VA received formal instruction about telemedicine technology and basic telemedicine concepts during the prior academic year, but widespread adoption of telemedicine remained limited before the COVID-19 pandemic.

We designed the telemedicine case-based discussion to fit into the regularly scheduled 45-minute preclinical conference series (PCC). Faculty preceptors lead small-group sessions for the weekly PCC, which follows the Yale curriculum for outpatient medicine.[20] To limit cognitive load for faculty preceptors, who were also novice telemedicine practitioners adjusting to the

Copyright © 2025 Jones et al. This is an open-access publication distributed under the terms of the Creative Commons Attribution license.

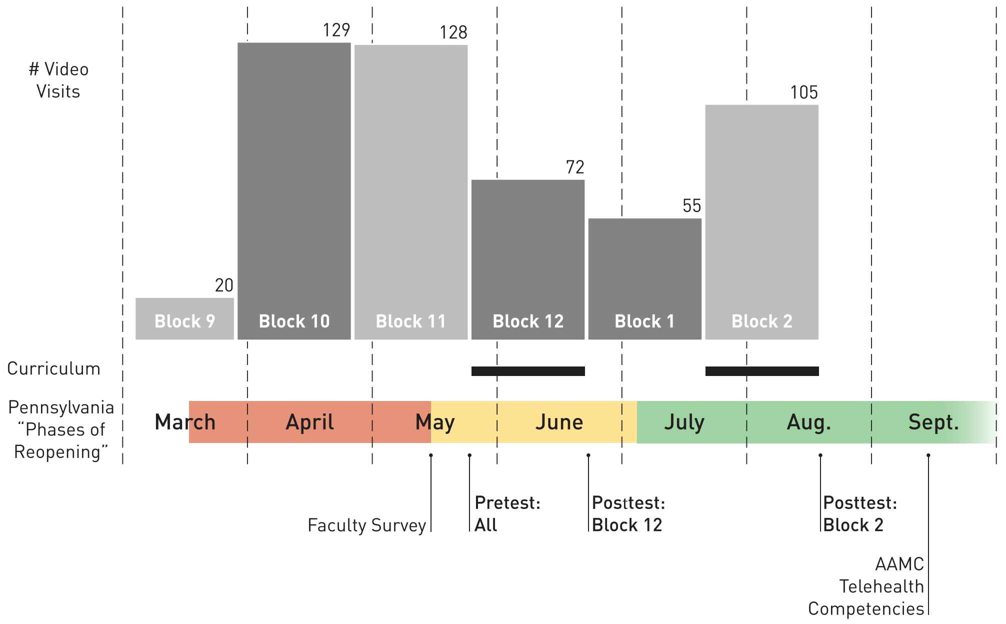

Figure. Telemedicine curriculum development and implementation. Video visit volume for internal medicine (IM) residents from EpicCare-based clinics is shown per block/continuity clinic rotation. Light gray–shaded and dark gray–shaded columns indicate the two cohorts of IM residents. Thick black horizontal lines indicate time of curriculum administration (May–June and July–August 2020). Given its likely influence on video visit volume, the color-coded system of red, yellow, and green is shown to represent the Phases of Reopening based on Pennsylvania public health guidance in response to the COVID-19 pandemic. Faculty needs assessment survey, pre- and postsession survey administration to residents, and preprint publication of the AAMC telehealth competencies are also noted on the time line.

pandemic, the case-based discussion format was intentionally modeled on the Yale curriculum style.

Needs Assessment

In May 2020, after 2 months of resident telemedicine experience, we conducted a needs assessment of continuity clinic faculty, who precepted residents at least 4 hours per week. Since no existing framework for teaching telemedicine skills to residents was available at the time, the authors used RedCap to develop a brief needs assessment survey of residents' telemedicine skills. The survey was sent via email to preceptors, and the response rate was 61% (28/46). Faculty prioritized three topics for a telemedicine curriculum: triaging patients to appropriate visit types; telemedicine communication skills; and virtual physical examination. Faculty reported directly observing 27% of in-person visits, compared with 16% of telemedicine video visits and 12% of telemedicine phone visits.

Curriculum Design

We developed a two-part telemedicine curriculum that involved a case-based discussion followed by formative direct observation, which is congruent with recommendations on the use of formal instruction to develop telemedicine skills of the novice.[21] Case-based curricula have been successful in telemedicine education.[10-12,15] Direct observation of telemedicine skills aligns with Miller's model for the optimal assessment of clinical competency through observation of behaviors in practice, is associated with positive educational outcomes, and is recommended for telemedicine skills assessments.[22,23]

Residents first participated in a 45-minute case-based discussion focused on patient triage, telemedicine communication skills, and physical exam adaptation, which was facilitated by faculty preceptors during designated PCC time. The faculty facilitator guide (Appendix A) mirrors the resident hand-out (Appendix B) and provides instructions for the case-based discussion, a framework for telemedicine visits, and suggested responses for discussion.

Within the case-based discussion, a patient triage framework developed by the authors was shared. The cognitive steps started with identifying patient safety issues for the patient's presenting concern and then anticipating how an in-person exam would change evaluation of that concern. The final step was to conclude which visit options, including phone, video, and in-person, were appropriate for the patient. Facilitators guided residents to apply this framework to cases described in the handout.

Nested within continuation of the third case, residents participated in a role play. One resident practiced adapting the shoulder exam to a video encounter on a peer, the patient,

Copyright © 2025 Jones et al. This is an open-access publication distributed under the terms of the Creative Commons Attribution license.

while other residents shared observations and suggestions. Descriptions of exam maneuvers were included, as the content focus was not the shoulder exam but rather adaptation of the physical exam to telemedicine.

The third and fourth cases provided discussion about telemedicine communication, such as defining the concept of webside manner and presenting a published strategy for managing interruptions and agenda setting.[24] These cases also included appropriate telemedicine follow-up and re-triage if a video visit was not appropriate.

The cases are summarized in the facilitator guide, which presents the skills practiced, the educational objectives, and the AAMC telehealth domains (Appendix A).

For the second part of the curriculum, faculty aimed to observe each resident perform one telemedicine visit over the next month. Video visit observation was preferred, but phone visit observation was acceptable. Faculty utilized a direct observation checklist, which was developed by the study authors in accordance with direct observation best practices, to deliver formative feedback to the resident immediately following the observed encounter or before conclusion of the clinic session (Appendix C).[25] Additionally, the checklist included one item used for an overall assessment of preceptor confidence in the quality of care delivered by the resident during the observed visit (Appendix C).

Faculty Development

Two weeks before delivering the curriculum, faculty preceptors attended a 45-minute faculty development session (Appendix D), over Zoom, that introduced the curriculum, case-based discussion facilitator guide, and direct observation checklist. Faculty guides included an optional reference to a brief Stanford telemedicine physical exam video example, if faculty desired additional preparation before delivering the curriculum (Appendix A).[26]

Curriculum Evaluation

Curriculum participants completed pre- and postsession surveys electronically, with presession surveys administered immediately before the curriculum and postsession surveys administered before residents rotated off the 4-week ambulatory block (Figure). We did not identify any existing, validated surveys for assessing the relevant telehealth domains in residents. Thus, question content was drawn from surveys used to evaluate medical school telemedicine curricula and from the results of our faculty needs assessment.[27]

On the pre- and postsession surveys (Appendix E), residents used 5-point Likert scales to rate their degree of efficiency with video and phone telemedicine visits compared to in-person visits (1 = *much more efficient*, 5 = *much less efficient*), level of agreement with attitudes toward telemedicine (1 = *strongly disagree*, 5 = *strongly agree*), and level of competence with 15 telemedicine skills (1 = *not competent*, 5 = *extremely competent*). Surveys were piloted with IM fellows for clarity and administered via RedCap. Responses were anonymous. No incentive was offered.

To assess experience with video visits prior to and during curriculum implementation, video visit counts were determined for each resident at EpicCare-based clinics between March and August 2020. Video visits were defined as visits scheduled for video and billed with appropriate video visit codes. Video visit counts were not available from the VA.

Data Analysis

Baseline demographic characteristics of participants were summarized descriptively using frequencies and percentages for all residents who completed the presession survey as well as for residents who completed both pre- and postsession surveys. Among those with complete outcome data, responses to agreement, efficiency, and competency questions were summarized as mean (SD) confidence ratings. To identify significant changes in these measures from pre- to postsession, we performed paired t tests. To determine if a difference in volume of video visits existed between the two resident groups, a two-sample t test was performed, with results expressed as mean (*SD*). A Type 1 error rate of 5% was used to establish the level of statistical significance, and no adjustments were made for multiplicity. All statistical analyses were performed using STATA/SE version 16.1 (College Station, TX).

Institutional Review and Ethical Approval

The Quality Improvement Review Committee at the University of Pittsburgh (Project ID #2694) provided project oversight.

Results

Of the 152 eligible residents, 119 residents (78%) participated in the curriculum. Among them, 80% of residents (95/119) completed the presession survey, while 51% (61/119) completed both the pre- and postsession surveys. Characteristics of these groups were similar and are shown in Table 1. Before curriculum participation, 28% of residents reported prior training and 13% reported prior hands-on experience with telemedicine (Table 1). All residents reporting prior training and experience completed an introductory VA telemedicine curriculum during the previous academic year.

Copyright © 2025 Jones et al. This is an open-access publication distributed under the terms of the Creative Commons Attribution license.

Table 1. Characteristics of Residents Participating in the Presession Survey (N = 95) or Pre- and Postsession Surveys (N = 61)

Characteristic	Presession Survey *n* (%)	Both Pre- and Postsession Survey *n* (%)
Year of Training		
PGY 1	29 (31)	19 (31)
PGY 2	35 (37)	28 (46)
PGY 3	25 (26)	11 (18)
N/A	6 (6)	3 (5)
Gender		
Male	40 (42)	31 (51)
Female	45 (47)	26 (43)
Prefer not to answer	7 (7)	4 (7)
N/A	3 (3)	0 (0)
Site		
Montefiore	35 (37)	25 (41)
Shadyside	15 (16)	10 (16)
Birmingham	3 (3)	2 (3)
South Hills	4 (4)	4 (7)
VA	35 (37)	19 (31)
N/A	6 (6)	3 (5)
Training		
Yes	29 (31)	17 (28)
No	59 (62)	40 (66)
N/A	7 (7)	4 (7)
Experience		
Yes	10 (11)	8 (13)
No	79 (83)	50 (82)
N/A	6 (6)	3 (5)
Group		
May–June 2020	42 (44)	28 (46)
July–August 2020	47 (49)	30 (49)
N/A	6 (6)	3 (5)

Abbreviations: PGY, postgraduate year; N/A, not applicable; VA, Veterans Administration.

On the presession surveys, residents expressed confidence in their efficiency with telemedicine and positive attitudes, and no improvements in self-perceived efficiency with telemedicine or in attitudes toward telemedicine were observed after the session (Table 2). Residents reported achieving a higher degree of efficiency with telemedicine visits than with in-person visits. Residents agreed that telemedicine was useful for managing patients' acute and chronic concerns but felt the quality of in-person visits was better than video visits (Table 2). Residents reported interest in using telemedicine in future practice.

Self-perceived competence with all surveyed telemedicine skills increased between the presession and postsession surveys (Table 3). In the EpicCare-based clinics, residents in the first group (May–June 2020) and second group (July–August 2020) had conducted a mean of 3.9 video visits (SD = 2.3) and 4.9 video visits (SD = 2.4; p = .22), respectively, before curriculum completion. By the time they had completed the curriculum, residents in the first group and second group had conducted a mean of 6.2 video visits (SD = 2.4) and mean of 8.4 video visits (SD = 3.8; p = .06), respectively. Improvement in competence was independent of level of training for all residents completing the curriculum. Moreover, among residents with the EpicCare-based clinics, competence was independent of video visit volume (data not reported).

Overall, 61% of residents (73/119) received direct observation. Within the first curriculum group, 34% of residents (23/68) received direct observation feedback on a video visit and 18% of residents (12/68) received direct observation feedback on a phone visit. Among the second curriculum group, 75% of residents (38/51) received direct observation feedback on a video visit; no observations were completed for phone visits. Thirty faculty preceptors completed these direct observations.

On 86% of direct observations (63/73), preceptors rated the skills of most residents as *performed well*, whereas they rated one or more skills of the residents as *needs improvement* on 14% of directly observed resident visits (10/73). Specifically, preceptors identified with equal frequency use of effective nonverbal communication skills, ability to overcome communication stuck points, effective time management, and ability to perform appropriate physical exam given the patient's concerns and within constraints of the video encounter as a skill that most often needed improvement (14%, 2/14 observed skills needing improvement). Preceptors rated their level of confidence in care delivered by residents as follows: *very confident* (82%, 60/73), *moderately confident* (15%, 11/73), *slightly confident* (1%, 1/73), or *not confident* (1%, 1/73).

Discussion

Following participation in this two-part telemedicine curriculum using case-based discussion followed by direct observation, IM residents reported improved self-perceived competence with telemedicine skills independent of resident level of training or video visit volume. Largest gains were observed in triaging patients to visit type, performing virtual physical exam, addressing preventative needs, and arranging for follow-up after telemedicine visits. Reassuringly, after direct observation, many faculty preceptors expressed confidence in the telemedicine care provided by residents. Residents expressed a relatively high level of self-perceived competence in telemedicine skills even before participating in the curriculum, suggesting that many skills used for face-to-face medical care can translate to video visits. The skills with the most improvement were those specific to telemedicine, such as triage to visit type and arranging follow-up testing after the video visit. Notably, on the presession

Copyright © 2025 Jones et al. This is an open-access publication distributed under the terms of the Creative Commons Attribution license.

Table 2. Changes in Residents' Self-Perceived Efficiency With and Attitudes Toward Telemedicine for All Paired Presession–Postsession Surveys (N = 61)

Survey Item	Pre *M (SD)*	Post *M (SD)*	Delta *M (SD)*	*p*
E1. How would you rate your efficiency with telemedicine video visits compared with in-person visits?[a]	3.6 (1.1)	3.8 (0.8)	0.2 (0.9)	.10
E2. How would you rate your efficiency with telemedicine phone visits compared with in-person visits?[a]	4.0 (0.9)	3.7 (1.1)	−0.3 (1.1)	.04[b]
A1. Telemedicine has utility for managing patients' acute concerns.[c]	3.9 (0.9)	3.9 (0.9)	0.1 (1.0)	.51
A2. Telemedicine has utility for managing patients' chronic conditions.[c]	4.5 (0.6)	4.6 (0.5)	0.0 (0.6)	.50
A3. The quality of telemedicine visits is the same as in-person visits.[c]	2.6 (1.0)	2.7 (1.0)	0.1 (0.9)	.58
A4. I would like to use telemedicine in my future practice.[c]	4.2 (0.9)	4.3 (0.8)	0.1 (0.6)	.40

[a]Rated on a 5-poiint Likert scale (1 = *much more efficient*, 5 = *much less efficient*).
[b]$p < .05$.
[c]Rated on a 5-point Likert scale (1 = *strongly disagree*, 5 = *strongly agree*).

survey, residents felt the least competent in performing the virtual physical exam, and residents reported significant gains in self-perceived competence with this skill on the postsession survey.

Strengths of this curriculum include its quick, easy implementation into existing educational time and scalability across multiple clinic sites when rapid adoption of telemedicine demanded effective telemedicine education.[6] Additionally, the curriculum responded to a local needs assessment, prioritizing needs similar to those identified on a contemporary telemedicine OSCE, including three areas for resident competency development: technical proficiency, information gathering (including history and physical examination), and communication skills.[28] Our residents had hands-on experience with telemedicine technology and reported feeling moderately to very competent with these skills, and therefore our curriculum prioritized teaching patient triage skills, given our local needs assessment. The curriculum has utility in the postpandemic era, as telemedicine remains widely used and is a competency for physicians across the spectrum of practice and for IM residents.[8,9]

Our curriculum presents lessons and opportunities for improvement. Given the novelty of telemedicine care in 2020, we conducted a 45-minute session for faculty development. However, most faculty are now generally experienced in telemedicine, and therefore faculty development could be abbreviated or shifted to independent study. For curriculum delivery, rapid implementation during the early pandemic limited participation to only 80% of eligible residents. Many residents were unable to attend as they covered patient care needs across the program. Additionally, not all participants received direct observation because video visit volumes fluctuated. Nonurgent curriculum delivery should improve these challenges. Interestingly, although direct observations were overwhelmingly positive—potentially suggesting that the checklist was an inadequate tool—residents viewed direct observation feedback as meaningful, which may indicate that some in-person skills are transferrable to telemedicine skills with dedicated focus from

Table 3. Changes in Residents' Self-Perceived Competence With Telemedicine Skills for All Paired Presession–Postsession Surveys (N = 61)

Telemedicine Skill[a]	Pre *M (SD)*	Post *M (SD)*	Delta *M (SD)*	*p*
C1. Determining when a patient concern is appropriate for a telemedicine visit versus in-person visit	3.1 (0.7)	3.6 (0.6)	0.5 (0.7)	<.001[b]
C2. Managing patients' acute concerns during telemedicine visits	3.0 (0.7)	3.3 (0.7)	0.3 (0.7)	<.001[b]
C3. Managing patients' chronic conditions during telemedicine visits	3.6 (0.7)	3.9 (0.6)	0.3 (0.6)	<.001[b]
C4. Determining plan to address patients' preventative care needs during telemedicine visits	3.1 (0.9)	3.6 (0.9)	0.5 (0.9)	<.001[b]
C5. Using technology to connect with a patient over audio or video for a telemedicine visit	3.7 (0.9)	3.9 (0.8)	0.2 (0.8)	.02[c]
C6. Collecting history through telemedicine	4.0 (0.8)	4.3 (0.7)	0.3 (0.7)	.002[b]
C7. Performing physical exam through telemedicine	2.0 (0.7)	2.5 (0.8)	0.5 (0.7)	<.001[b]
C8. Counseling patients through telemedicine on the plan	3.8 (0.8)	4.1 (0.7)	0.3 (0.7)	.001[b]
C9. Using the electronic health record during telemedicine visits (includes placing orders, writing notes, billing)	3.6 (1.0)	3.9 (0.9)	0.2 (0.9)	.04[c]
C10. Presenting a patient evaluated through telemedicine to your clinic preceptor	3.7 (0.8)	4.0 (0.7)	0.3 (0.8)	.002[b]
C11. Arranging for patient follow-up testing after a telemedicine visit	3.3 (0.9)	3.8 (0.8)	0.5 (0.9)	<.001[b]
C12. Setting an agenda at the beginning of a telemedicine visit	3.5 (0.7)	3.7 (0.8)	0.2 (0.8)	.03[c]
C13. Establishing an effective rapport with a patient during a telemedicine visit	3.5 (0.7)	3.9 (0.8)	0.4 (0.6)	<.001[b]
C14. Redirecting the conversation with a patient if needed during a telemedicine visit	3.3 (0.7)	3.7 (0.8)	0.3 (0.8)	.003[b]
C15. Effective time management during a telemedicine visit	3.3 (0.7)	3.7 (0.8)	0.4 (0.8)	<.001[b]

[a]Rated on a 5-point Likert scale (1 = *not competent*, 5 = *extremely competent*).
[b]$p < .01$.
[c]$p < .05$.

Copyright © 2025 Jones et al. This is an open-access publication distributed under the terms of the Creative Commons Attribution license.

the case-based discussion. Previously defined best practices for direct observations can be implemented to ensure future success, such as designating faculty champions to encourage direct observation and normalizing a programmatic culture of direct observation for formative feedback.[25]

For curriculum evaluation, the response rate among participants who completed both the pre- and postsession survey was 51%. Offering an incentive for survey completion and nonurgent curriculum implementation may improve response rates. PGY 3 residents had the lowest response rate for pre- and postsession surveys. Curriculum timing coincided with residency graduation for the first group of PGY 3 residents, and with fellowship applications for the second group. The 4-week postsession survey captured resident responses before they rotated off ambulatory blocks or graduated, but a longer interval to postsession follow-up could evaluate for durability of improvement in telemedicine skill confidence. Additionally, we evaluated this curriculum with self-reported competence rather than an objective skill evaluation, due to the time-sensitive need for telemedicine education. Although a telemedicine OSCE suggested residents were overconfident in telemedicine-specific skills when compared to standardized patients' assessments of observed behaviors, educators expressed concern that lack of resident confidence prevented full utilization of telemedicine.[29,30] Therefore, without validated skill evaluation options at the time of curriculum implementation, improvements in self-perceived competence were a reasonable outcome to assess.

Another limitation is that although resident confidence with visit triage increased, nursing staff often perform visit triage. Additionally, we did not capture video visit volume for IM residents at the VA clinic; therefore we were unable to evaluate the relationship between visit volume and survey results at this clinic site. Finally, our curriculum evaluation was not controlled. Although residents may have increased self-perceived competence due to maturation effect, our analysis demonstrated that these improvements were independent of resident level of training at all sites and independent of video visit volume for residents with EpicCare-based clinics.

In conclusion, this two-part telemedicine curriculum for IM residents uniquely aligns with three AAMC telehealth competency domains through case-based discussion with a novel framework for telemedicine triage combined with direct observation. The need for telemedicine education continues, given widespread use of telemedicine and formalized expectations for physician competency in telemedicine. As telemedicine clinical care and technology evolve, this easy-to-implement curriculum can be expanded or adapted to cover additional AAMC competency domains and to address emerging learner needs, including residents in IM or other specialties and even advanced medical students with basic telemedicine experience.

Appendices

A. Faculty Facilitator Guide.docx

B. Resident Handout.docx

C. Direct Observation Checklist.docx

D. Telehealth Faculty Development Session.pptx

E. Pre- and Posttest.docx

All appendices are peer reviewed as integral parts of the Original Publication.

Sarah Jones, MD, MS: Assistant Professor, Division of General Internal Medicine, University of Pittsburgh School of Medicine; ORCID: https://orcid.org/0000-0002-8108-7026

Melissa McNeil, MD, MS: Professor and Clinician Educator, Department of Medicine, The Warren Alpert Medical School of Brown University

Scott D. Rothenberger, PhD: Assistant Professor and Associate Director for Early-Career Collaborations, Center for Research on Health Care Data Center, Department of Medicine, University of Pittsburgh School of Medicine

Kwonho Jeong, MS: Statistician, Department of Medicine, University of Pittsburgh School of Medicine

Tanya Nikiforova, MD, MS: Associate Professor and Associate Program Director for Ambulatory Education, Division of General Internal Medicine, University of Pittsburgh School of Medicine

Acknowledgments

The authors thank Nicholas Rebeck for designing the figure in the publication, Emily Joseph for extracting video visit data from EpicCare, and Ethan Lennox for manuscript editing.

Disclosures

None to report.

Funding/Support

Dr. Sarah Jones received a Division of General Internal Medicine Clinician Educator Award from Medical Center, University of Pittsburgh.

Prior Presentations

Jones S, Nikiforova T. Adapting education to a new era: tips for teaching in telehealth encounters. Presented at: Enhancing Teaching Skills for Medical Educators: A Lunchtime Learning Webinar; September 2020; Pittsburgh, PA.

Copyright © 2025 Jones et al. This is an open-access publication distributed under the terms of the Creative Commons Attribution license.

Jones S, Nikiforova T. Adapting education to a new era: tips for telemedicine encounters with learners. Presented at: University of Pittsburgh Faculty Development Meeting; November 2020; Pittsburgh, PA.

Jones S, McNeil M, Nikiforova T. Training internal medicine residents to perform high-quality telemedicine visits: a novel skill-based curriculum. Plenary presentation at: University of Pittsburgh Department of Medicine Research Day; April 2021; Pittsburgh, PA.

Jones S, McNeil M, Nikiforva T. Training internal medicine residents to perform high-quality telemedicine visits: a novel skill-based curriculum. *J Gen Intern Med*. 2021;36(suppl 1):S462. Abstracts from the 2021 Annual Meeting of the Society of General Internal Medicine. https://doi.org/10.1007/s11606-021-06830-5

Ethical Approval

The Quality Improvement Review Committee at the University of Pittsburgh reviewed this project.

References

1. Flodgren G, Rachas A, Farmer AJ, Inzitari M, Shepperd S. Interactive telemedicine: effects on professional practice and health care outcomes. *Cochrane Database Syst Rev*. 2015; 2015(9):CD002098. https://doi.org/10.1002/14651858.CD002098.pub2
2. Ohta M, Ohira Y, Uehara T, et al. How accurate are first visit diagnoses using synchronous video visits with physicians? *Telemed J E Health*. 2017;23(2):119-129. https://doi.org/10.1089/tmj.2015.0245
3. Harju A, Neufeld J. Telehealth utilization during the COVID-19 pandemic: a preliminary selective review. *Telemed Rep*. 2022; 3(1):38-47. https://doi.org/10.1089/tmr.2021.0040
4. Chandrasekaran R. Telemedicine in the post-pandemic period: understanding patterns of use and the influence of socioeconomic demographics, health status, and social determinants. *Telemed J E Health*. 2024;30(2):480-489. https://doi.org/10.1089/tmj.2023.0277
5. Pourmand A, Ghassemi M, Sumon K, Amini SB, Hood C, Sikka N. Lack of telemedicine training in academic medicine: are we preparing the next generation? *Telemed J E Health*. 2021;27(1): 62-67. https://doi.org/10.1089/tmj.2019.0287
6. Stovel RG, Gabarin N, Cavalcanti RB, Abrams H. Curricular needs for training telemedicine physicians: a scoping review. *Med Teach*. 2020;42(11):1234-1242. https://doi.org/10.1080/0142159X.2020.1799959
7. Sartori DJ, Olsen S, Weinshel E, Zabar SR. Preparing trainees for telemedicine: a virtual OSCE pilot. *Med Educ*. 2019;53(5): 517-518. https://doi.org/10.1111/medu.13851
8. *New and Emerging Areas in Medicine Series: Telehealth Competencies Across the Learning Continuum*. Association of American Medical Colleges; 2021. Accessed June 3, 2025. https://www.google.com/url?sa=t&source=web&rct=j&opi=89978449&url=https://store.aamc.org/downloadable/download/sample/sample_id/412/&ved=2ahUKEwiawd3h7tWNAxVQF1kFHY-mOBYQFnoECBsQAQ&usg=AOvVaw1ncD8M8PEsAOv1
9. *Internal Medicine Milestones*. Accreditation Council for Graduate Medical Education. Updated November 2020. Accessed June 3, 2025. https://www.acgme.org/globalassets/PDFs/Milestones/WxgxR3RDInternalMedicineMilestones.pdf
10. Mulcare M, Naik N, Greenwald P, et al. Advanced communication and examination skills in telemedicine: a structured simulation-based course for medical students. *MedEdPORTAL*. 2020;16:11047. https://doi.org/10.15766/mep_2374-8265.11047
11. Cornes S, Gelfand JM, Calton B. Foundational telemedicine workshop for first-year medical students developed during a pandemic. *MedEdPORTAL*. 2021;17:11171. https://doi.org/10.15766/mep_2374-8265.11171
12. Cantone RE, Palmer R, Dodson LG, Biagioli FE. Insomnia telemedicine OSCE (TeleOSCE): a simulated standardized patient video-visit case for clerkship students. *MedEdPORTAL*. 2019; 15:10867. https://doi.org/10.15766/mep_2374-8265.10867
13. McDaniel LM, Molloy M, Hindman DJ, et al. Phone it in: a medical student primer on telemedicine consultation in pediatrics. *MedEdPORTAL*. 2021;17:11067. https://doi.org/10.15766/mep_2374-8265.11067
14. Wilson LA, Harder B, Kelley C, Powell R, Foster M, Roberts E. Geriatric telehealth: a standardized patient case for medical students. *MedEdPORTAL*. 2023;19:11345. https://doi.org/10.15766/mep_2374-8265.11345
15. Wang SK, Goodrich RC, Strauss LD, Martindale JM. High-yield, case-based, interactive workshop on telehealth and teleneurology with pediatric resident physicians. *MedEdPORTAL*. 2023;19:11340. https://doi.org/10.15766/mep_2374-8265.11340
16. Kirkland EB, DuBose-Morris R, Duckett A. Telehealth for the internal medicine resident: a 3-year longitudinal curriculum. *J Telemed Telecare*. 2021;27(9):599-605. https://doi.org/10.1177/1357633X19896683
17. Savage DJ, Gutierrez O, Montane BE, et al. Implementing a telemedicine curriculum for internal medicine residents during a pandemic: the Cleveland Clinic experience. *Postgrad Med J*. 2022;98(1161):487-491. https://doi.org/10.1136/postgradmedj-2020-139228
18. McConnell M, Kumar P, Mullur R. The virtual visit: a telehealth curriculum for internal medicine residents featuring a virtual physical examination. *J Grad Med Educ*. 2023;15(5):582-586. https://doi.org/10.4300/JGME-D-22-00635.1
19. Ha E, Zwicky K, Yu G, Schechtman A. Developing a telemedicine curriculum for a family medicine residency. *PRiMER*. 2020;4:21. https://doi.org/10.22454/PRiMER.2020.126466

Copyright © 2025 Jones et al. This is an open-access publication distributed under the terms of the Creative Commons Attribution license.

20. Yale office-based medicine curriculum. Yale School of Medicine. Accessed June 3, 2025. https://medicine.yale.edu/intmed/obm
21. Pathipati AS, Azad TD, Jethwani K. Telemedical education: training digital natives in telemedicine. *J Med Internet Res*. 2016;18(7):e193. https://doi.org/10.2196/jmir.5534
22. Miller GE. The assessment of clinical skills/competence/performance. *Acad Med*. 1990;65(9)(suppl):S63-S67. https://doi.org/10.1097/00001888-199009000-00045
23. Noronha C, Lo MC, Nikiforova T, et al. Telehealth competencies in medical education: new frontiers in faculty development and learner assessments. *J Gen Intern Med*. 2022;37(12):3168-3173. https://doi.org/10.1007/s11606-022-07564-8
24. Mauksch LB. Questioning a taboo: physicians' interruptions during interactions with patients. *JAMA*. 2020;323(17):1704-1705. https://doi.org/10.1001/jama.2020.2980
25. Andolsek KM, Simpson D. Direct observation reassessed. *J Grad Med Educ*. 2017;9(4):531-532. https://doi.org/10.4300/JGME-D-17-00328.1
26. Srinivasan M, Artandi M. How to administer a virtual physical exam. Standford Medicine Department of Medicine News. Accessed June 3, 2025. http://medicine.stanford.edu/news/current-news/standard-news/virtual-physical-exam.html
27. Walker C, Echternacht H, Brophy PD. Model for medical student introductory telemedicine education. *Telemed J E Health*. 2019;25(8):717-723. https://doi.org/10.1089/tmj.2018.0140
28. Lawrence K, Hanley K, Adams J, Sartori DJ, Greene R, Zabar S. Building telemedicine capacity for trainees during the novel coronavirus outbreak: a case study and lessons learned. *J Gen Intern Med*. 2020;35(9):2675-2679. https://doi.org/10.1007/s11606-020-05979-9
29. Sartori DJ, Hayes RW, Horlick M, Adams JG, Zabar SR. The TeleHealth OSCE: preparing trainees to use telemedicine as a tool for transitions of care. *J Grad Med Educ*. 2020;12(6):764-768. https://doi.org/10.4300/JGME-D-20-00039.1
30. Samberg D, Jones S, Nikiforova T, Norman M, McNamara M. Best practices for resident telemedicine training—a toolkit for success. Workshop presented at: Society of General Internal Medicine Annual Meeting; April 2021; Boston, MA.

Received: December 18, 2024
Accepted: June 2, 2025
Published: July 8, 2025

Copyright © 2025 Jones et al. This is an open-access publication distributed under the terms of the Creative Commons Attribution license.
